# The Dark Side of Ideology: Ideological Worldviews and Antidemocratic Attitudes

**DOI:** 10.1111/nyas.70062

**Published:** 2025-09-27

**Authors:** Artur Nilsson, Ali Teymoori

**Affiliations:** ^1^ Department of Psychosocial Science University of Bergen Bergen Norway; ^2^ Department of Behavioural Sciences and Learning Linköping University Linköping Sweden

**Keywords:** authoritarian, autocracy, democracy, extremism, ideology, totalitarian

## Abstract

This research investigated associations between diverse aspects of ideological worldviews and opposition to principles of liberal democracy in a heterogeneous sample of UK adults (*N* = 824). In line with the hypotheses, both system‐justifying worldviews (e.g., authoritarianism and social dominance orientation) and system‐challenging worldviews (e.g., need for chaos and prejudice against the rich and powerful) were robustly associated with antidemocratic attitudes, adjusting for other predictors and demographic variables—and these associations were mediated by perceived illegitimacy of the democratic system. Several system‐orthogonal aspects of worldviews, including a simplistic epistemology (e.g., lack of actively open‐minded thinking) and misperceptions of antidemocratic attitudes among political opponents, also robustly predicted antidemocratic attitudes, while political prejudice, societal discontent, and perceived superiority or the self and ingroup showed less robust effects. At a more specific level, the strongest predictors of support for democratic elections, censorship, political violence, and denying groups their rights were actively open‐minded thinking, authoritarianism, need for chaos, and social dominance orientation, respectively. Taken together, the findings contribute to a more unified and nuanced understanding of the psychological underpinnings of antidemocratic attitudes.

## Graphical abstract

This study investigated associations between ideological worldviews and opposition to liberal democracy in a UK sample (*N* = 824). System‐justifying (e.g., authoritarianism and social dominance) and system‐challenging (e.g., need for chaos and prejudice against the rich) worldviews robustly predicted antidemocratic attitudes, mediated by perceived illegitimacy of the democratic system. System‐orthogonal aspects of worldviews, including a simplistic epistemology and misperceptions of antidemocratic attitudes among political opponents, also had unique effects.

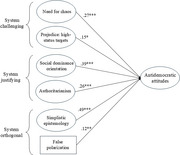

## 1 Introduction

Ideology has a profound impact on human life. It has undoubtedly played a prominent role in the establishment of liberal democratic institutions, which has led to a more peaceful world. At the same time, it harbors tremendous potential for fostering division and destruction—indeed, it is responsible for some of the worst atrocities in human history [1]—and enthusiasm about the triumph of liberal democracy has today been tempered by alarm reports about democratic backsliding all over the world [2] and a gradual erosion of democratic values even in established democracies [3]. An understanding of the psychological underpinnings of antidemocratic tendencies in the contemporary world is, therefore, urgently needed.

Although there has recently been a substantial upsurge of research on this topic, the literature is highly fragmented, encompassing a multitude of isolated research programs on factors such as authoritarianism, dominance motivations, political animosity, false polarization, need for chaos, conspiracy theories, sense of deprivation, and political cynicism [4, 5, 6, 7, 8, 9, 10]. There is a need for a broader synthesis of scholarship on antidemocratic tendencies [11]. As a first step, the goal of the current research was to expand and integrate the field by cross‐sectionally testing and comparing the most prominent explanations extracted from the literatures on democratic values, authoritarianism, extremism, and radicalization—explanations that, in one way or another, focus on the individual's worldview.

To adequately capture the kind of ideological thinking that might underlie antidemocratic tendencies, it is, we contend, not sufficient to focus merely on left‐right or liberal‐conservative asymmetries at the surface‐level (e.g., in terms of self‐placement, identity, or issue preferences)—rather, a richer understanding of the worldviews underlying ideological thought is needed. Broadly defined, ideologies not only describe the proper order of society; they also, as Jost et al. [12, p. 309] put it, “describe or interpret the world as it is—by making assertions or assumptions about human nature, historical events, present realities, and future possibilities”—that is, they are embedded in broader worldviews [13, 14]. On this conceptualization, assumptions about, for example, the deterioration of society, the superiority of the ingroup, the evilness of human nature, the trustworthiness of authorities, and even the moral depravity of political opponents may be part of the individual's ideological universe.

### 1.1 Antidemocratic Attitudes

Studies have used a multitude of measures focusing on, for example, general democratic values based on items included in large panel studies [6, 15], support for representative, direct, deliberative, and other types of democracy [10], and willingness to tradeoff democratic principles for other values [16]. Some have focused on more specific themes, such as support for illiberal policies [17], political violence [18], and constitutional protections [19], or on multiple dimensions, such as free and fair elections, legal and political equality, and defection from democratic norms [8, 20].

Constructing a single comprehensive measure of antidemocratic attitudes may be difficult, as there is no consensus on what constitutes democracy. Although democracy, in its elementary form, means government *by* and *for* the people, this encompasses a wide range of meanings across different theories, contexts, and historical periods [21]. Theories of democracy (e.g., electoral, deliberative, liberal, egalitarian, and participatory democracy) differ in terms of what is placed at the core of democracy—for example, fair and competitive elections, presence of elected executive officials, protection of various liberties (e.g., expression, association, or participation), inclusion of diverse social groups, and universal suffrage. In political science, a fruitful way to manage this complexity has been to focus on multiple indicators on which there is consensus across democratic theories, rather than relying on a simpler unidimensional characterization of democracy as the opposite of dictatorship [21].

In this vein, Claassen et al. [22] developed a new measure of support for liberal democracy based on the Varieties of Democracy (V‐dem) project, which is the most comprehensive attempt by political scientists to conceptualize and measure democracy across the world [2, 21]. Instead of asking participants abstract questions about support for democratic or authoritarian regimes, it measures support for the eight specific principles of the V‐dem definition of liberal democracy: freedom of expression, freedom of association, universal suffrage, free and fair elections, key decision‐makers being elected, equality before the law, and judicial and legislative constraints on the executive branch of government. Results from 19 countries supported a unidimensional model of this measure, although some items (e.g., protest rights, judicial review, and technocratic rule) exhibited low factor loadings in less developed contexts [22].

In spite of its strengths, a limitation of this measure is that it does not distinguish any subdimensions. An alternative multidimensional model was recently introduced based on a review of the literature on democratic attitudes in political psychology. Nilsson [11] proposed that there are four major dimensions covering attitudes unequivocally opposed to the principles of liberal democracy across previous measures of antidemocratic attitudes. These include support for the elimination or weakening of democratic institutions, such as free elections; violent action against the democratic system to advance a political cause (e.g., insurgencies or terrorism); suppression of criticism and ideas inconsistent with one's beliefs (e.g., persecuting journalists or censoring dissent); and discrimination against specific groups through denial of their democratic rights (e.g., political participation and equal treatment under the law).

In the current research, we treated these two approaches—a top‐down approach based on political science [22] and a bottom‐up psychometric approach [11]—as complementary. Thus, we measure both a general dimension of attitudes opposed to liberal democracy and four subdimensions covering attitudes toward elections, violence, censorship, and discrimination.

### 1.2 Ideological Worldviews

One of the most fundamental differences between ideologies is whether they justify and bolster the status quo or seek to challenge and overthrow it [12]. This difference is reflected in antidemocratic predispositions [11]. System‐justifying ideologies can be expected to lead to antidemocratic tendencies insofar as they increase the probability that people will defer to an authoritarian leader in order to protect the social order and the conventional norms from perceived threats. System‐challenging ideologies, on the other hand, can be expected to lead to antidemocratic tendencies insofar as they increase the probability that people will be willing to sacrifice or overthrow democratic principles when they feel that their superordinate ideological values are being violated or threatened. These are two fundamentally different routes to antidemocratic tendencies [11].

There are also aspects of ideological worldviews that are conceptually orthogonal to attitudes about the system but may nevertheless lead to antidemocratic attitudes. They may do so by a variety of routes, such as putting the individual in a mindset that entails susceptibility to totalitarian ideas and propaganda or by fueling partisan animosity and misperceptions about political opponents.

#### 1.2.1 System‐Justifying Worldviews

The theory of authoritarianism [23] may be the most classic account in this area. It rose to prominence after World War II by providing an explanation for why many people had been willing to subordinate themselves to totalitarian leaders. It did so by identifying a set of psychological characteristics—an “authoritarian syndrome”—hypothesized to entail a susceptibility to fascist propaganda. The original list was diverse, including characteristics such as rigidity, stereotypical thinking, and cynicism that fit better into other categories than the system‐justifying worldviews. However, the conceptualization subsequently became narrower and more homogeneous, focusing on three dimensions: *submission* to socially sanctioned authorities in positions of power, *aggression* sanctioned by the government toward those who threaten or deviate from conventional norms, and a *conventionalism* involving adherence to established authority‐sanctioned norms and conventions—characteristics that are associated with a tendency to justify and bolster the current system [24].

A voluminous body of research has shown that authoritarianism is associated with prejudice against marginalized groups and a willingness to justify political violence, sacrifice civil liberties and human rights, and restrict criticism of the government [5, 9, 17, 23] (for a review, see Ref. [25]). Most recently, De Oliveira Santos and Jost [8] found that authoritarianism in the United States was associated with willingness to vote for an antidemocratic candidate and to restrict political equality and legal rights and guarantees; justification of political violence and defection from democratic rules; and political intolerance.

Interestingly, some studies [8, 16] have found that antidemocratic attitudes are even more strongly associated with social dominance orientation (SDO)—a construct initially developed to explain group‐based inequality (e.g., racism, sexism, and classism), intergroup conflict, and the oppression and discrimination of low‐status groups [26]. SDO is a particularly powerful predictor of generalized prejudice against derogated groups (e.g., the poor, ethnic minorities, and gay people), while authoritarianism better predicts prejudice against groups that are perceived as dangerous [25]. De Oliveira Santos and Jost [8] found that authoritarianism was a better predictor of willingness to defect from democratic rules, but only SDO uniquely predicted willingness to vote for an antidemocratic candidate as well as lower support for political equality and legal rights and guarantees; an exception to these patterns was that both constructs predicted *higher* support for free speech. Furthermore, Bartusevičius et al. [6] found that both SDO and risk‐taking in the pursuit of dominance and status predicted preference for autocracy (vs. democracy) and political violence in South Africa, Denmark, and the United States.

#### 1.2.2 System‐Challenging Worldviews

Some researchers have criticized the theory of authoritarianism for focusing mainly on characteristics thought to make people susceptible to totalitarian ideologies on the right. Most recently, Costello et al. [27] developed a scale to measure parallel characteristics that occur among radical liberals in the United States, including *aggression* directed at the rich and powerful at the top of the social hierarchy, *censorship* of ideas perceived as offensive or ideologically incongruent, and an *anti‐conventionalism* involving absolutism about progressive values and intolerance of other values. Although this conceptualization illuminates relevant aspects of system‐challenging worldviews, it overlaps significantly with antidemocratic attitudes, including support for political violence and censorship [11], which would make tests of the role of system‐challenging worldviews in antidemocratic attitudes circular. Another problem is that it covers a diverse set of characteristics—while some are truly system‐challenging (e.g., prejudice against the rich), others are more properly treated as system‐orthogonal (e.g., political prejudice and group‐based authoritarianism) and measured across the ideological spectrum. Following Nilsson's proposal [11], we therefore sought to more clearly isolate different components from each other and from antidemocratic attitudes.

A key ingredient in system‐challenging worldviews is some form of dissatisfaction with the current order. At an early stage, this may involve a perception of grievance or deprivation—for example, of a person or group being unfairly treated and deprived of its rightful position—fueling outrage, contempt, and hostility that may culminate in political violence [5, 28, 29, 30]. It may also involve a more generalized sense of discontent and cynicism with respect to the current state of affairs and the political system, portraying society as unjust or corrupt (e.g., fascist, woke, or perverted)—and, in the extreme case, apocalyptic narratives that describe the current situation as catastrophic and as leading us toward destruction unless radical measures are taken [30, 31]. In particular, the sense that the current democratic system is illegitimate is an important element in radical ideologies of different kinds and may serve as an important precursor to the rejection of democratic principles [5, 10, 32]. This may in turn be rooted in a perceived breakdown of the social fabric (i.e., an erosion of moral values and trust) and the political leadership—that is, a sense of *anomie*, which can lead to rioting, extremism, and political instability when it becomes widespread in society [33].

System‐challenging worldviews may also be nourished by what has been termed a *need for chaos*, involving a desire to disrupt order and destroy established structures [4]. This desire may, when it is combined with a sense of deprivation or marginalization, lead to a motivation to overthrow the system, either to subvert the power hierarchy or to replace the existing system with something better. It is indeed strongly associated with support for political violence [34].

#### 1.2.3 System‐Orthogonal Worldviews

Black‐and‐white thinking has often been identified as one of the key components of extremism, radicalism, and authoritarianism that recurs across political ideologies and religions, including both system‐justifying and system‐challenging worldviews [27, 30, 32, 35]. This type of mindset, which involves seeing the world in terms of rigid dichotomies, may be particularly destructive insofar as it entails a tribalist us‐versus‐them mentality [20, 29, 32] or, in the more extreme case, a Manichean picture of the world as a battle between the good, righteous, and pure and the evil, corrupt, and filthy [18, 30]. A black‐and‐white mindset may, furthermore, entail a belief in simple solutions to societal problems [10] and a utopian vision of a paradise or perfect world (e.g., a classless or ethnically cleansed nation) [30, 31], without acknowledging nuances, practical complications, or strengths and weaknesses of different kinds of social arrangements.

A related aspect of extremist thinking is the perception of personal or ingroup superiority. This may involve epistemic overconfidence [35], perceptions of moral superiority [32], or a messianic sense of special significance or being chosen [30]. Such perceptions of superiority might undermine the tolerance for a plurality of opinions (e.g., results of elections) required in a democracy [15], making people feel entitled to impose their beliefs on others through coercion and violence. Furthermore, the need to feel significant, and experiences that involve loss of significance (e.g., feeling humiliated or demeaned), may play an important role in the radicalization process [36].

Another contributing factor is what might be called a post‐truth mentality, through which facts, rational discourse, and legitimate expertise are replaced with subjective opinions, rhetoric, dogmatism, and tribalism [37, 38, 39]. This mentality plays a role in antidemocratic tendencies that are system‐justifying, insofar as authoritarian leaders use propaganda, misinformation, indoctrination, and delegitimization of professions that pursue the truth (e.g., academics, journalists, and judges) to replace objective truth with their fictional narratives [9]. It may also play a key role in system‐challenging tendencies, particularly in today's digital world, where people can fall into rabbit holes full of conspiracy theories about the system and other alternative narratives, bolstered by like‐minded peers and a contempt for official sources [5, 10].

Finally, political animosity and polarization may contribute to antidemocratic attitudes across the ideological spectrum. Some studies have suggested that affective polarization (i.e., antipathy toward political opponents) is associated with antidemocratic attitudes [19], while others have suggested that merely disliking political opponents has limited effects [18, 40], and that more extreme forms of sectarianism, involving hatred, dehumanization, and perceptions of evil, matter more [30, 34, 41]. There is also recent evidence of effects driven by false polarization—people are more willing to sacrifice democratic principles and values when they falsely believe that their political opponents are willing to do the same [7, 20].

All these system‐orthogonal worldview elements do not necessarily comprise one single coherent worldview—for example, post‐truth relativism and dogmatic black‐and‐white thinking may represent different epistemological extremes, albeit with similarly pernicious consequences [38]. Nevertheless, a common element that in one way or another recurs across many of the system‐orthogonal characteristics is a simplistic and uncritical epistemology, including a failure to recognize limitations, nuances, and complexities (e.g., with respect to the self, others, morality, knowledge, or politics), similar to the mindset documented among extremists, involving rigidity, narcissism, stereotypical thinking, and demonization of opponents [27, 30, 35].

### 1.3 Overview of Research

This research tested a series of preregistered hypotheses, based on the foregoing theoretical considerations, stating that the following variables would be associated with antidemocratic attitudes (for more details, see the preregistration):

1. System‐justifying worldviews: (a) authoritarianism, (b) dominance motivation, and (c) prejudice against powerless groups,
2. System‐challenging worldviews: (a) need for chaos, (b) pessimism about society and deprivation, and (c) prejudice against powerful groups,
3. System‐orthogonal worldviews: (a) black‐and‐white thinking, (b) post‐truth attitude, (c) perceived superiority of self and ingroup, and (d) prejudice against political opponents and false perceptions of antidemocratic attitudes among them.

Additionally, we hypothesized that the effects of system‐justifying and system‐challenging predictors (excluding prejudice) would be mediated by perceived legitimacy and illegitimacy of the system, respectively. More specifically, we expected that individuals with system‐justifying worldviews would be more tolerant of infractions of democratic principles because of a desire to defend and legitimize the government [11, 24], whereas individuals with system‐challenging worldviews would be more willing to turn against the democratic system insofar as they perceive it as illegitimate (e.g., rigged or manipulated) [11, 32].

The tests of hypotheses were followed up by regression analyses and structural equation modeling exploring which predictors had the strongest and most unique effects over and above the effects of other predictors. In the regression analyses, we also investigated the robustness of effects adjusting for demographic variables (gender, age, education, left‐right placement, and political and religious activity). Furthermore, we adjusted for a generally negative outlook on the world, including cynicism about human nature and belief that the world is dangerous, which may be more related to system‐justifying ideology [13, 25] compared to cynicism about society, and we similarly adjusted for group‐based authoritarianism [42], which can be seen as more compatible with various forms of ideology compared to traditional authoritarianism.

## 2 Materials and Methods

Preregistration,^1^ materials, data, and supplemental information about measures and analyses^2^ are openly accessible.

The research was carried out in compliance with relevant regulations concerning research ethics. It did not involve any experimental manipulation or collection of personally identifiable information and is, therefore, not covered by the Swedish law regarding ethical review of research on humans. The participants were informed about the purposes and procedures of the research and provided their informed consent to participate.

### 2.1 Participants

UK adults recruited through Prolific completed a survey administered through Qualtrics. The sample was stratified by age and education so that it contained equal proportions of participants over/under 40 years old and with/without higher education. Nine participants who had failed preregistered exclusion criteria were excluded. The remaining sample contained 824 participants (45.1% men, 54.0% women; Age, *M* = 41.3; *SD* = 14.1). Of these, 805 had completed all scales.

### 2.2 Materials

The study was divided into eight sections, presented to participants on separate pages. Item order was randomized within the first six sections. These sections are shown in Table 1 in the order in which the participants completed them, along with information about the scales they included. Scales of similar types were grouped together within sections. Attention check items were included in Sections 2, 4, and 6. The participants responded on Likert scales ranging from 1 (“Strongly disagree”) to 7 (“Strongly agree”). Complete sources and details are available in the Supporting Information: https://osf.io/y4qra.

**TABLE 1:** Sections and scales included in the survey along with numbers of items, reliability statistics, and sample items.

Sections and scales	Items	Reliability			Sample item
		*α*	*ω*_h_	*ω*_t_	
Section 1					
V‐dem score^a^	15	0.80	0.51	0.83	“People should be free to criticize the government even in times of great crisis.”
Elections	5	0.82	0.74	0.84	“A political system without parties and elections can be preferable when people vote for the wrong politicians.”
Censorship	5	0.74	0.61	0.80	“Political messages from some groups should be blocked or censored from the public.”
Discrimination	4	0.63	0.61	0.66	“It is more important that the laws protect our country's interests than that all individuals have the same rights to one hundred percent.”
Violence	5	0.86	0.84	0.88	“Political violence can be constructive when it serves the right cause.”
Total index^b^	31	0.90	0.71	0.92	
Section 2					
Social dominance orientation (SDO)	8	0.87	0.75	0.90	“Some groups of people are simply inferior to other groups.”
Authoritarian submission	4	0.69	0.64	0.77	“We should believe what our leaders tell us.”
Authoritarian aggression	4	0.61		0.65	“It is necessary to use force against people who are a threat to authority.”
Group‐based authoritarianism	3	0.69		0.71	“A group member should always obey group rules.”
Section 3					
Breakdown of social fabric	4	0.69	0.67	0.71	“People think that there are no clear moral standards to follow.”
Group deprivation	4	0.84	0.84	0.87	“I think that people in my group are disadvantaged because society oppresses them.”
Individual deprivation	5	0.82	0.76	0.85	“Others are to blame for the problems people like myself face.”
Apocalypticism	4	0.85	0.83	0.86	“Today the human race is on the edge of an enormous calamity.”
Meritocratic world	4	0.85	0.84	0.86	“People who do their job well rise to the top.”
Cynicism	4	0.80	0.78	0.81	“Human beings tend to exploit others.”
Dangerous world	4	0.79	0.74	0.84	“Basically, this world is generally a dangerous place.”
Section 4					
System legitimacy	6	0.88	0.87	0.93	“The government uses its power legitimately.”
System illegitimacy	5	0.88	0.79	0.90	“We live in a sham democracy where a small group secretly controls the country.”
System justification	6	0.83	0.75	0.86	“Everyone has a fair shot at wealth and happiness.”
Distrust in politicians	4	0.81	0.79	0.84	“Politicians don't care about the problems of the average person.”
Section 5					
Dichotomous knowledge	4	0.74	0.64	0.82	“Most problems have only one right answer.”
Manicheanism	4	0.88	0.87	0.89	“Life is a battle between good and evil.”
Utopianism	5	0.85	0.75	0.89	“With the right vision, a near‐perfect society could be constructed.”
Subjectivist relativism	5	0.89	0.84	0.90	“Truth is a subjective feeling; if it feels correct or obvious to a person, then it is true.”
Conspiracist ideation	4	0.88	0.88	0.89	“Politicians and other leaders are nothing but the string puppets of powers operating in the background.”
Distrust in experts	4	0.84	0.84	0.85	“Those who are called experts are generally overrated.”
Actively open‐minded thinking (AOT)	5	0.72	0.41	0.80	“Opinions should always be revised in response to new information or evidence.”
Section 6					
Need for chaos	7	0.81	0.65	0.85	“Sometimes I just feel like destroying beautiful things.”
Status‐driven risk taking	3	0.71		0.73	“I would enjoy being a famous and powerful person, even if it meant a high risk of assassination.”
Grandiosity	6	0.88	0.82	0.91	“I'm better than almost everyone else.”
Collective narcissism	5	0.82	0.75	0.84	“My group deserves special treatment.”
Intellectual overconfidence	5	0.80	0.70	0.83	“My ideas are usually better than other people's ideas.”

^a^ V‐dem score: The Claassen et al. [22] measure.

^b^ Total index: Based on all antidemocratic items loading on the general factor [11, 22]. The hierarchical omega coefficient is omitted for scales with too few items to yield a meaningful estimate.

After the first six sections, the participants completed a section with scales measuring prejudice and false polarization. They completed the same six items for low‐ and high‐status groups (“poor and powerless [rich and powerful] people at the bottom [top] of society”; *α* ≥ 0.91, *ω*_h_ ≥ 0.79, *ω*_t_ ≥ 0.95) and for least‐ and most‐liked party (“people who vote for the political party you like the least [the most], that is your political opponents [political allies]”; *α* ≥ 0.93, *ω*_h_ ≥ 0.81, *ω*_t_ ≥ 0.96), rating these groups on six attributes such as “evil” and “savage” on Likert scales ranging from 1 (“Not at all”) to 7 (“Extremely”). They also indicated how they thought people who vote for the parties they like the least (*α* = 0.80, *ω*_h_ = 0.61, *ω*_t_ = 0.84) and the most (*α* = 0.74, *ω*_h_ = 0.59, *ω*_t_ = 0.80) would respond to five of the antidemocratic attitude items. For participants who reported one of the six largest parties as their least‐liked, we computed a misperception (i.e., false polarization) score by subtracting actual responses of participants from this group (i.e., who chose this party as their most‐liked) from perceived responses of the least‐liked group on the same items. An analogous misperception score was computed for the most‐liked party.

A final section of the survey measured demographics, including gender, age, level of education, left‐right self‐placement (1 = “Very far to the left” to 9 = “Very far to the right”; *M* = 4.51, *SD* = 1.69), liberal‐conservative self‐placement (1 = “Extremely liberal” to 9 = “Extremely conservative”; *M* = 4.51, *SD* = 1.77), most‐ and least‐liked parties, religiosity (denomination and level), and the degree to which the participant was politically active (*M* = 35.7, *SD* = 26.7 on a 0–100% scale).

### 2.3 Statistical Procedure

Confirmatory factor analyses ascertained the distinctness of the scales. The measurement models generally exhibited adequate fit (https://osf.io/y4qra and https://osf.io/fesxm), and the reliability coefficients were, in most cases, excellent (Table 1). Hierarchical and total omega coefficients and Cronbach's alpha were computed in the psych package in R (v. 4.4) [43].

For tests of hypotheses, we report correlations with 95% bias‐corrected bootstrap confidence intervals (10,000 resamples). Although we preregistered sequential Holm−Bonferroni corrections to adjust for testing multiple hypotheses, this proved unnecessary because all hypothesized correlations except one yielded *p*‐values below 0.001. We ran four separate hierarchical regression models: (1) system‐justifying predictors; (2) system‐challenging predictors; (3) system‐orthogonal predictors; and (4) all predictors. In all models, demographic variables (male vs. female gender, age, education, left‐right placement, level of religiosity, and political activity) were entered in a separate block; the second regression model also contained a separate block including cynicism and dangerous‐world beliefs. We ran mediation analyses using the PROCESS macro in SPSS (v. 29) [44]. We ran these analyses both with system legitimacy and system illegitimacy as parallel mediators and with a system legitimacy score aggregated across both scales (*α* = 0.90, *ω*_h_ = 0.69, *ω*_t_ = 0.93) as a single mediator; we report results based on the parallel mediation tests to provide a clearer and more nuanced picture. We report standardized indirect effects with 95% bootstrap confidence intervals (10,000 resamples). All these analyses followed the preregistered plan.

We used structural equation modeling conducted in AMOS 29.0 (maximum likelihood estimation) to further probe the robustness of the effects. The predictors were represented as latent factors (except false polarization, which was manifest) with items as indicators. Second‐order factors were included to account for the strong associations between predictors: authoritarianism (submission, aggression, and group‐based; *λ* ≥ 0.56), societal discontent (perceived breakdown of the social order, relative deprivation, and apocalypticism; *λ* ≥ 0.50), prejudice (high‐status, low‐status, and political targets; *λ* ≥ 0.55), narcissism (grandiosity, collective narcissism, intellectual overconfidence; *λ* ≥ 0.79), and simplistic epistemology (black‐and‐white and post‐truth predictors; *λ* ≥ 0.63). Covariances were allowed between predictors. Antidemocratic attitudes were initially represented as a latent factor with the four subdimensions as indicators, but we also ran separate models investigating effects on the subdimensions. Complete details are provided in the Supporting Information: https://osf.io/fesxm.

## 3 Results

Correlations between antidemocratic attitudes and predictors, mediators, and demographic variables are shown in Table 2.

**TABLE 2:** Zero‐order correlations between system‐justifying, system‐challenging, system‐orthogonal, and demographic predictors and antidemocratic attitudes.

	Antidemocratic attitudes					
	Total index	V‐dem score	Elections	Censorship	Discrimination	Violence
System‐justifying predictors						
SDO	0.53***	0.48***	0.37***	0.26***	0.62***	0.19***
Status‐driven risk taking	0.26***	0.19***	0.27***	0.10**	0.18***	0.22***
Authoritarian submission	0.34***	0.36***	0.23***	0.32***	0.23***	0.11**
Authoritarian aggression	0.44***	0.36***	0.30***	0.27***	0.46***	0.30***
Group‐based authoritarianism	0.38***	0.36***	0.37***	0.23***	0.30***	0.13***
Prejudice: low‐status targets	0.36***	0.29***	0.30***	0.11**	0.27***	0.27***
System legitimacy	−0.13***	−0.06	−0.24***	0.17***	0.01	−0.16***
System justification	0.01	0.06	−0.09*	0.19***	0.13***	−0.12***
System‐challenging predictors						
Need for chaos	0.46***	0.35***	0.42***	0.13***	0.27***	0.43***
Breakdown of social fabric	0.32***	0.25***	0.37***	0.05	0.20***	0.22***
Group‐based deprivation	0.24***	0.17***	0.29***	0.05	0.04	0.23***
Individual deprivation	0.31***	0.22***	0.34***	0.05	0.19***	0.24***
Apocalypticism	0.13***	0.07*	0.16***	0.07*	0.07*	0.12***
Cynicism	0.18***	0.15***	0.26***	0.02	0.08*	0.06
Meritocratic world (reversed)	−0.10**	−0.14***	−0.11**	−0.13***	−0.11**	0.08*
Dangerous world belief	0.16***	0.16***	0.22***	−0.01	0.11**	0.00
Distrust in politicians	0.21***	0.13***	0.32***	−0.02	0.12***	0.14***
Prejudice: high‐status targets	0.29***	0.18***	0.28***	0.07*	0.08*	0.37***
System illegitimacy	0.40***	0.31***	0.47***	0.06	0.19***	0.26***
System‐orthogonal predictors						
Dichotomous epistemology	0.49***	0.44***	0.44***	0.23***	0.41***	0.23***
Manicheanism	0.28***	0.25***	0.32***	0.12***	0.20***	0.08*
Utopianism	0.45***	0.40***	0.46***	0.21***	0.24***	0.24***
Subjectivism	0.36***	0.34***	0.40***	0.14***	0.26***	0.08*
Conspiracist ideation	0.40***	0.31***	0.46***	0.06	0.22***	0.27***
Distrust in experts	0.40***	0.35***	0.42***	0.07	0.34***	0.15***
AOT (reversed)	0.55***	0.53***	0.50***	0.25***	0.41***	0.19***
Collective narcissism	0.33***	0.26***	0.33***	0.13***	0.21***	0.23***
Grandiosity	0.42***	0.33***	0.34***	0.21***	0.35***	0.29***
Intellectual overconfidence	0.45***	0.36***	0.41***	0.17***	0.41***	0.25***
Political prejudice	0.23***	0.13***	0.18***	0.06	0.05	0.34***
Misperception: least‐liked	0.19***	0.13***	0.14***	0.10*	0.08*	0.25***
Misperception: most‐liked	0.59***	0.50***	0.50***	0.35***	0.41***	0.39***
Demographics						
Female (vs. male) gender	0.07*	0.14***	0.10**	0.08*	−0.03	−0.09**
Age	−0.15***	−0.13***	−0.17***	0.00	0.15***	−0.25***
Left‐right position	0.30***	0.31***	0.25***	0.07	0.42***	−0.01
Liberal‐conservative position	0.35***	0.35***	0.31***	0.09**	0.45***	0.03
Religiosity	0.15***	0.16***	0.14***	0.07*	0.05	0.06
Political activity	−0.03	−0.09*	−0.08*	−0.05	−0.02	0.18***

* *p* < 0.005; ** *p* < 0.01; *** *p* < 0.001.

### 3.1 System‐Justifying Ideology

Consistent with our hypotheses, the aggregated antidemocratic index correlated strongly with SDO [0.47, 0.58], status‐driven risk taking [0.18, 0.34], authoritarian submission [0.26, 0.41], authoritarian aggression [0.38, 0.50], group‐based authoritarianism [0.31, 0.44], and prejudice against low‐status targets [0.29, 0.43] (95% BcA CI; *p* < 0.001). The correlations generally held up across the four subdimensions, although their strengths varied (Table 2).

Together, the hypothesized predictors accounted for 38.4% of the variance in antidemocratic attitudes (43.7% with demographic variables; tolerance ≥ 0.59). As shown in Table 3, SDO had the strongest unique effect of the system‐justifying predictors, followed by aggression, submission, group authoritarianism, status‐driven risk taking (*p* ≤ 0.006), and prejudice against low‐status targets (*p* = 0.020), and the regression coefficients remained robust when demographic variables were included.

**TABLE 3:** Standardized regression coefficients (with 95% confidence intervals) based on separate models for each group of predictors and an integrated model with all predictors.

	Separate models		Integrated model	
	1. Predictors	2. Demographics	1. Predictors	2. Demographics
System‐justifying predictors				
SDO	0.32 [0.25, 0.38]***	0.34 [0.27, 0.40]***	0.24 [0.17, 0.31]***	0.24 [0.16, 0.32]***
Status‐driven risk taking	0.09 [0.01, 0.03]**	0.08 [0.02, 0.14]**	−0.02 [−0.08, 0.05]	−0.01 [−0.08, 0.06]
Authoritarian submission	0.15 [0.09, 0.21]***	0.14 [0.08, 0.22]***	0.14 [0.07, 0.21]***	0.11 [0.04, 0.18]**
Authoritarian aggression	0.20 [0.14, 0.27]***	0.22 [0.15, 0.28]***	0.21 [0.14, 0.28]***	0.22 [0.15, 0.28]***
Group‐based authoritarianism	0.09 [0.03, 0.16]**	0.10 [0.04, 0.16]**	0.01 [−0.06, 0.08]	0.02 [−0.05, 0.09]
Prejudice: low‐status targets	0.07 [0.01, 0.14]*	0.06 [0.00, 0.12]	−0.03 [−0.10, 0.04]	−0.03 [−0.11, 0.04]
System‐challenging predictors				
Need for chaos	0.34 [0.27, 0.41]***	0.31 [0.24, 0.38]***	0.12 [0.04, 0.20]**	0.13 [0.05, 0.21]**
Breakdown of social fabric	0.14 [0.06, 0.21]***	0.16 [0.07, 0.24]***	0.02 [−0.07, 0.10]	0.01 [−0.07, 0.10]
Group‐based deprivation	−0.02 [−0.11, 0.07]	0.01 [−0.08, 0.10]	0.05 [−0.04, 0.15]	0.03 [−0.06, 0.13]
Individual deprivation	0.09 [0.00, 0.19]	0.08 [−0.01, 0.18]	−0.05 [−0.14, 0.04]	−0.04 [−0.13, 0.05]
Apocalypticism	−0.05 [−0.12, 0.02]	0.00 [−0.08, 0.07]	0.05 [−0.02, 0.12]	0.06 [−0.01, 0.13]
Cynicism	−0.10 [−0.19, −0.01]*	−0.09 [−0.18, 0.00]*	−0.05 [−0.14, 0.03]	−0.06 [−0.15, 0.02]
Dangerous‐world belief	0.02 [−0.06, 0.10]	−0.04 [−0.12, 0.04]	−0.07 [−0.15, 0.00]	−0.08 [−0.16, −0.01]*
Meritocratic world (reversed)	−0.19 [−0.25, −0.12]***	−0.13 [−0.19, −0.07]***	−0.05 [−0.11, 0.02]	−0.04 [−0.10, 0.02]
Distrust in politicians	−0.01 [−0.09, 0.07]	−0.05 [−0.12, 0.03]	0.01 [−0.07, 0.09]	−0.02 [−0.10, 0.06]
Prejudice: high‐status targets	0.12 [0.05, 0.19]***	0.15 [0.08, 0.22]***	0.13 [0.04, 0.21]**	0.12 [0.03, 0.20]**
System‐orthogonal predictors				
Dichotomous epistemology	0.15 [0.07, 0.23]***	0.14 [0.06, 0.22]***	0.04 [−0.04, 0.12]	0.04 [−0.04, 0.12]
Manicheanism	−0.07 [−0.15, 0.00]	−0.07 [−0.15 0.01]	−0.09 [−0.17, −0.02]*	−0.07 [−0.15, 0.00]
Utopianism	0.14 [0.06, 0.22]***	0.15 [0.07, 0.23]***	0.12 [0.05, 0.19]***	0.12 [0.05, 0.19]***
Subjectivism	0.02 [−0.06, 0.09]	0.01 [−0.07, 0.08]	0.05 [−0.02, 0.12]	0.04 [−0.03, 0.11]
Conspiracist ideation	0.10 [0.01, 0.18]*	0.08 [−0.01, 0.16]	0.10 [0.02, 0.19]*	0.09 [0.01, 0.17]*
Distrust in experts	0.09 [0.01, 0.17]*	0.06 [−0.03, 0.14]	0.05 [−0.03, 0.13]	0.06 [−0.02, 0.13]
AOT (reversed)	0.30 [0.22, 0.38]***	0.27 [0.19, 0.36]***	0.22 [0.14, 0.29]***	0.21 [0.13, 0.28]***
Collective narcissism	−0.05 [−0.13, 0.04]	−0.05 [−0.14, 0.03]	−0.01 [−0.10, 0.08]	−0.02 [−0.11, 0.07]
Grandiosity	0.13 [0.04, 0.23]**	0.17 [0.07, 0.26]***	−0.04 [−0.13, −0.05]	0.00 [−0.09, 0.10]
Intellectual overconfidence	0.00 [−0.10, 0.10]	−0.01 [−0.11, 0.09]	−0.04 [−0.13, 0.06]	−0.03 [−0.12, 0.06]
Political prejudice	0.02 [−0.05, 0.09]	0.03 [−0.05, 0.10]	−0.02 [−0.11, 0.06]	−0.03 [−0.11, 0.05]
Misperception: least‐liked	0.14 [0.07, 0.21]***	0.14 [0.07, 0.20]***	0.10 [0.04, 0.16]**	0.10 [0.04, 0.16]**
Demographics				
Female (vs. male) gender				0.09 [0.03, 0.15]**
Age				−0.10 [−0.16, −0.03]**
Education				−0.04 [−0.10, 0.02]
Left‐right position				0.02 [−0.05, 0.09]
Religiosity				−0.02 [−0.08, 0.05]
Political activity				−0.08 [−0.14, −0.02]**

* *p* < 0.05; ** *p* < 0.01; *** *p* < 0.001.

Perceived system legitimacy and system justification failed to correlate positively with the antidemocratic index (Table 2). There were, therefore, no positive indirect effects of authoritarianism or SDO on general antidemocratic attitudes, contrary to our hypotheses. There were, however, positive indirect effects of authoritarian aggression and SDO on general antidemocratic attitudes through perceived system illegitimacy (Table 4). Furthermore, support for censorship correlated positively with perceived system legitimacy (Table 2), and an exploratory analysis revealed a positive indirect effect of authoritarian submission on support for censorship through perceived system legitimacy (0.074 [0.028, 0.122]).

**TABLE 4:** Standardized indirect effects on the general antidemocratic index through perceived system legitimacy and illegitimacy.

	Mediator 1: System legitimacy	Mediator 2: System illegitimacy
Authoritarian submission	−0.038 [−0.082, 0.004]	−0.054 [−0.085, −0.025]
Authoritarian aggression	0.004 [−0.003, 0.015]	0.054 [0.023, 0.085]
SDO	−0.004 [−0.015, 0.006]	0.062 [0.031, 0.092]
Need for chaos	−0.036 [−0.060, −0.015]	0.152 [0.111, 0.194]
Breakdown of social fabric	−0.064 [−0.098, −0.032]	0.203 [0.154, 0.253]
Group‐based deprivation	−0.053 [−0.085, −0.024]	0.206 [0.159, 0.255]
Individual deprivation	−0.058 [−0.089, −0.027]	0.198 [0.152, 0.244]
Distrust in politicians	−0.102 [−0.172, −0.030]	0.359 [0.295, 0.420]

### 3.2 System‐Challenging Ideology

Consistent with our hypotheses, the antidemocratic index correlated strongly with need for chaos [0.47, 0.58], perceived breakdown of the social fabric [0.18, 0.34], and prejudice against high‐status targets [0.23, 0.36] (*p* < 0.001) and these associations were highly robust adjusting for the other system‐challenging predictors, even when cynicism and dangerous‐world belief were added to the model (Table 3). Individual relative deprivation was also strongly correlated with antidemocratic attitudes [0.25, 0.37] (*p* < 0.001), but the effect was marginal, adjusting for the other predictors (*p* = 0.056; with cynicism and dangerous‐world belief, *p* = 0.037). Also consistent with the hypotheses, group‐based deprivation [0.17, 0.30], cynicism [0.11, 0.26], distrust in politicians [0.15, 0.28], and apocalypticism [0.06, 0.20] correlated with antidemocratic attitudes (*p* < 0.001), but none of these had unique effects adjusting for the other predictors (Table 3). Meritocratic beliefs correlated with antidemocratic attitudes in the opposite direction of what was hypothesized [0.02, 0.17] (*p* = 0.006; Tables 2 and 3). Taken together, the hypothesized predictors accounted for 25.2% of the variance (33.0% with demographic variables; tolerance ≥ 0.38).

Perceived system illegitimacy correlated strongly with need for chaos, relative deprivation, breakdown of the social fabric, distrust in politicians (*r* ≥ 0.44, *p* < 0.001), and antidemocratic attitudes (Table 2). The effects of need for chaos, relative deprivation, breakdown of social fabric, and distrust in politicians on antidemocratic attitudes were consistently mediated by perceived system illegitimacy (Table 3), supporting the hypotheses.

### 3.3 System‐Orthogonal Ideology

Consistent with the hypotheses, all variables measuring black‐and‐white thinking, post‐truth attitude, and perceived superiority correlated strongly with antidemocratic attitudes: dichotomous view of knowledge [0.44, 0.55], Manicheanism [0.21, 0.35], utopianism [0.39, 0.50], subjectivism [0.29, 0.42], lack of actively open‐minded thinking (AOT) [0.50, 0.60], distrust in experts [0.33, 0.46], conspiracist ideation [0.34, 0.46], collective narcissism [0.26, 0.39], grandiosity [0.35, 0.48], and intellectual overconfidence [0.38, 0.51] (*p* < 0.001). Prejudice against people who vote for the least‐liked party and misperceptions of their antidemocratic attitudes also correlated moderately with antidemocratic attitudes (Table 2). A follow‐up mixed linear model (not preregistered) showed an interaction effect between type of target (least‐/most‐liked) and prejudice on antidemocratic attitudes (*β* = 0.20 [0.13, 0.26], *p* < 0.001). Interestingly, misperceptions of antidemocratic attitudes among people who vote for the most‐liked party correlated even more strongly with the antidemocratic index (Table 2), but this is likely because people infer attitudes of their ingroup from their own attitudes.

The system‐orthogonal predictors together accounted for 41.6% of the variance in the antidemocratic index (45.0% with demographic variables; tolerance ≥ 0.37). Dichotomous view of knowledge, utopianism, low AOT, grandiosity, and outgroup misperceptions were the strongest unique predictors of the antidemocratic index (*p* < 0.001). Distrust in experts (*p* = 0.033) and conspiracism (*p* = 0.027) made marginal contributions that did not hold up adjusting for demographics (Table 3).

### 3.4 Integrated Model

All hypothesized predictors together accounted for 53.3% of the variance in the antidemocratic index (57.7% with demographic variables; tolerance ≥ 0.33). As shown in Table 3, the predictors accounting for most unique variation over and above other predictors were authoritarianism, SDO, need for chaos, prejudice against high‐status targets, utopianism, lack of AOT, and outgroup misperceptions.

**FIGURE 1:**
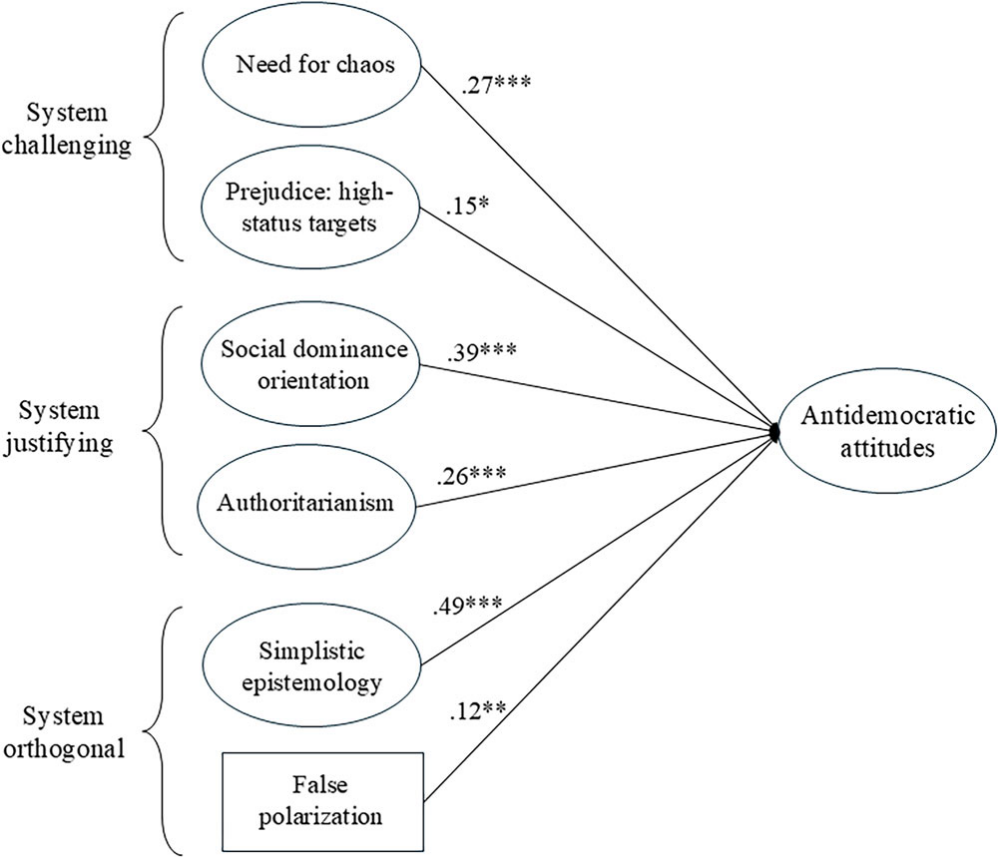
Standardized parameter estimates of effects on general antidemocratic attitudes based on structural equation modeling.

Structural equation modeling largely confirmed these results. This is illustrated in Figure 1, which shows predictors with significant unique effects on general antidemocratic attitudes. The model showed adequate fit in spite of its complexity, *χ* ^2^(6489) = 17,713 (*p* < 0.001), SRMR = 0.081, RMSEA = 0.046 [0.046, 0.047] (*λ* ≥ 0.24 for authoritarianism and 0.40 for all first‐order factors; *R* ^2^ = 81.6%). An even more complex model wherein all the predictors were separate (without common second‐order factors) suggested that effects of simplistic epistemology and authoritarianism were mainly accounted for by low AOT, conspiracist ideation, and authoritarian aggression.

**FIGURE 2:**
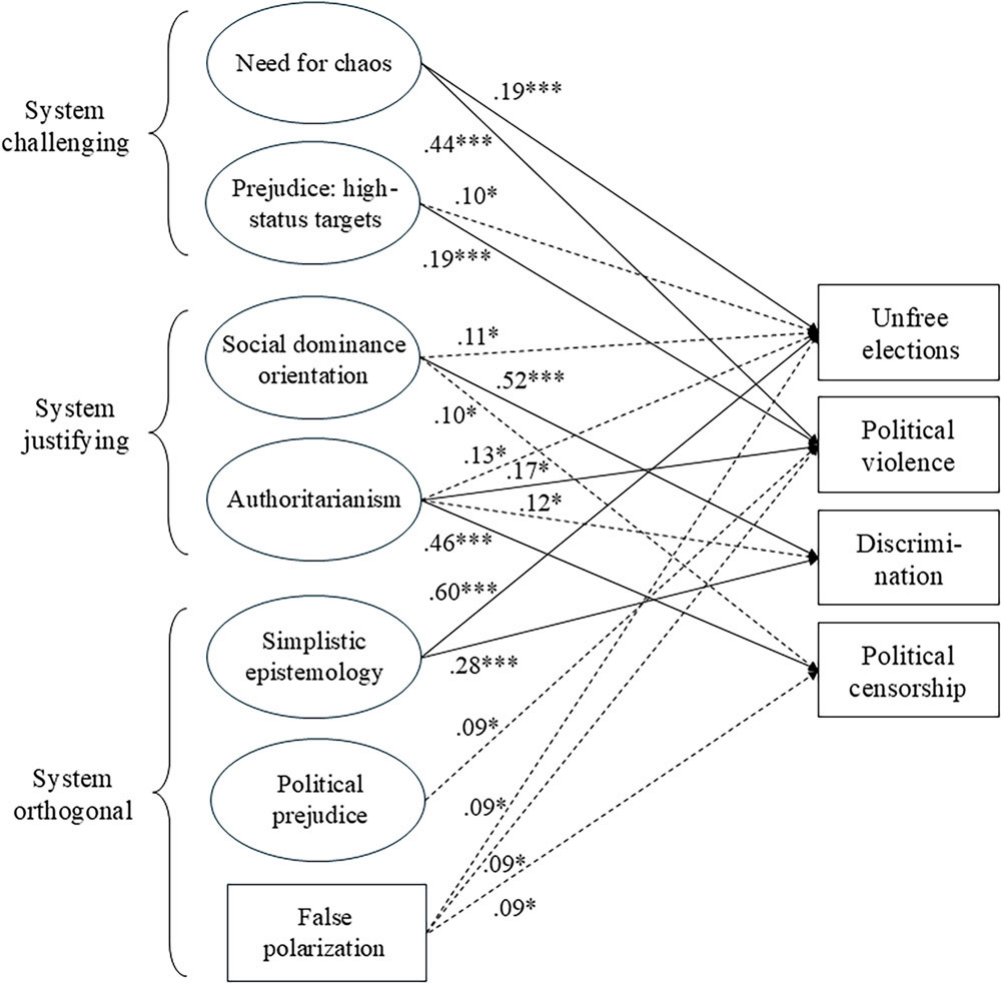
Standardized parameter estimates of effects on specific antidemocratic attitudes based on structural equation modeling. Dashed line: *γ* < 0.15.

Follow‐up analyses revealed substantial variation in effects when the four subdimensions were used as outcomes, as shown in Figure 2: democratic elections (*R* ^2^ = 47.4%), censorship (*R* ^2^ = 25.7%), discrimination (*R* ^2^ = 54.9%), and political violence (*R* ^2^ = 37.4%). The system‐justifying predictors of authoritarianism and SDO were by far the strongest unique predictors of the censorship and discrimination dimensions. By contrast, the system‐challenging predictors of need for chaos and prejudice against high‐status groups were (together with authoritarian aggression) the strongest unique predictors of support for political violence. Interestingly, while the system‐justifying and system‐challenging predictors had some unique effects on the democratic elections dimension too (see Figure 2), this dimension was most strongly predicted by a simplistic epistemology, including particularly low AOT and, to a lesser extent, conspiracist ideation and utopianism. Furthermore, AOT was, together with authoritarian aggression, the most consistent predictor overall, with substantial unique effects on the elections, censorship, and discrimination dimensions. Political prejudice and false polarization made marginal contributions, particularly to the prediction of support for political violence. For complete details, see the Supporting Information at https://osf.io/fesxm.

## 4 Discussion

This study investigated the association between ideological worldviews and antidemocratic attitudes in a diverse sample of UK adults. It is the first study in this area to be based on a broad, integrative framework encompassing a rich set of predictors from multiple research literatures (extremism, radicalization, democratic attitudes, and authoritarianism)—some of which had never before been investigated in this context—while also drawing on recent conceptual and psychometric advances [11, 22, 45]. It contributes to a more unified understanding of the psychological underpinnings of antidemocratic attitudes.

### 4.1 Findings

Nearly all hypotheses were supported by moderate to strong correlations. Furthermore, analyses comparing the predictors against each other showed that a diverse set of variables representing different categories of ideological worldviews contributed independently of each other to predicting antidemocratic attitudes. This is broadly consistent with the notion that there are multiple ideological routes to antidemocratic orientations.

#### 4.1.1 System‐Justifying and System‐Challenging Worldviews

Although there were stronger correlations between predictors within the categories of system‐justifying and system‐challenging predictors, there were also some positive correlations across the two categories. For example, need for chaos was associated with *higher* authoritarianism and SDO (see Supporting Information). These findings suggest that dark aspects of system‐justifying and system‐challenging worldviews—as operationalized in this study—share some common characteristics (e.g., aggressiveness), rather than representing polar opposites [11, 27].

In terms of system‐justifying worldviews, both authoritarianism and SDO stood out as unique and robust predictors across all models, which provides even stronger support for their role in antidemocratic orientations than previously found [8, 9, 16]. Authoritarian aggressiveness had a stronger and more robust effect than submission, suggesting that willingness to support violence matters more than obedience. Furthermore, the results suggest that traditional authoritarianism with respect to the conventional social order [23] matters more than group‐based authoritarianism [42].

Among the system‐challenging predictors, need for chaos and prejudice against high‐status (rich and powerful) targets stood out as the most robust ones. This provides novel evidence that these predictors are associated not only with support for political violence, as shown in prior research [27, 34], but also with antidemocratic attitudes more generally. It is possible that prejudice against high‐status groups plays a more unique role in system‐challenging totalitarianism [27] than prejudice against low‐status groups does in system‐justifying totalitarianism. Alternatively, this asymmetry may have emerged simply because authoritarianism and SDO, which are associated with prejudice against low‐status groups [25, 26], were included among the system‐justifying predictors. It is possible that prejudice against high‐status groups would also fail to have a unique effect if the battery of system‐challenging predictors included some other construct capable of accounting for this kind of prejudice without circularity—that is, a construct similar to left‐wing authoritarianism [27] but without direct overlap with prejudice against low‐status groups or antidemocratic attitudes.

In terms of societal discontent, the results suggest, consistent with the theory of anomie [33], that perceived breakdown of the social and moral fabric is the most important factor, trumping relative deprivation and distrust in politicians and people generally. It may be that experiences of deprivation play a role earlier in the radicalization process [29] and that they lead to antidemocratic attitudes only insofar as they are subsequently transformed into general discontent with society and the democratic system [32, 33], along with a desire to tear things down [4]. It is also possible, as results from structural equation modeling suggest, that even general societal discontent is associated with antidemocratic attitudes only by virtue of its overlap with other key predictors, such as conspiracist ideation and prejudice against high‐status targets. In line with this, apocalypticism—which was more independent of the other key predictors— was much more weakly correlated with antidemocratic attitudes. At the same time, the link between apocalypticism and antidemocratic attitudes may be contingent on perceived threats and contextual factors driving the apocalyptic beliefs (e.g., concern about global warming or multiculturalism).

#### 4.1.2 System‐Orthogonal Worldviews

The analyses also provided clear evidence that black‐and‐white and post‐truth mindsets are associated with antidemocratic attitudes independently of system‐justifying and system‐challenging worldviews. Interestingly, the predictors in both these categories shared a broad second‐order factor (“simplistic epistemology”), harking back to early conceptualizations that linked the authoritarian personality to simplistic and nonrational modes of thinking [23]. The most basic element of this factor appears to be a failure to rationally update beliefs based on new information—whether due to rigidity and intolerance of ambiguity or to trust in subjective hunches and alternative narratives—similar to lack of AOT [39]. Indeed, low AOT had both the highest loading on the second‐order factor in question and the most unique and robust effects on antidemocratic attitudes among the simplistic thinking styles (although conspiracist ideation also made a unique contribution). It was, in fact, among the best out of the entire battery of theory‐based predictors, even though it has rarely, if ever, been taken into consideration in this field. This is perhaps the most striking of all the findings, providing clear evidence that attitudes to knowledge and misinformation play a role in antidemocratic tendencies in today's world [9, 11]. Similarly, the results suggest, somewhat surprisingly, that a dichotomous view of knowledge plays a more important role than a Manichean view of good and evil. At the same time, utopian thinking about an ideal society was the most unique and robust predictor, particularly of willingness to restrict democratic elections, out of the black‐and‐white thinking styles, suggesting that it is an ingredient not just in militant extremism [30].

Grandiosity was the most unique predictor out of those measuring perceived superiority, suggesting that a sense of personal superiority is more important than group‐based superiority, which contradicts some previous findings [15]. Nevertheless, although grandiosity showed a unique effect beyond other system‐orthogonal predictors in this research, this did not hold up adjusting for system‐challenging and system‐justifying predictors. Grandiosity was, in fact, very strongly correlated with several other predictors, including SDO and need for chaos—both of which are associated with concerns about improving or upholding one's position in the status hierarchy, either by defending it [46] or by tearing it down [4]. In other words, grandiosity may be indirectly captured by other predictors.

Similarly, even blatant prejudice against political opponents did not uniquely predict general antidemocratic attitudes over and above the effects of other predictors. This is consistent with recent results that have cast doubt on the role of affective polarization in antidemocratic attitudes [18, 40], and it suggests that even stronger forms of antipathy against political opponents that are associated with antidemocratic attitudes [34, 41] for the most part fail to have a *unique* effect over and above other predictors. Instead, the results point to false polarization, involving misperceptions of antidemocratic attitudes among political opponents, playing a more decisive role, consistent with recent studies [7, 20].

#### 4.1.3 Subdimensions of Antidemocratic Attitudes

There were some variations across the four subdimensions of antidemocratic attitudes. The democratic elections dimension was the most strongly and consistently correlated with the predictors, exhibiting a pattern of correlations similar to the total antidemocratic score. The system‐justifying and system‐orthogonal predictors correlated consistently with the four subdimensions, but the system‐challenging predictors were largely uncorrelated with the censorship dimension (Table 2). Adjusting for all the best predictors, authoritarian aggression had the most consistent unique effects across the subdimensions, while the submission dimension only uniquely predicted support for censorship. SDO was the best predictor of the discrimination dimension, followed by authoritarian aggression, while need for chaos was the best predictor of support for political violence, followed by prejudice against high‐status groups. These findings align with our expectations regarding associations between discrimination and system‐justifying worldviews, as well as political violence and system‐challenging worldviews (see preregistration). Surprisingly, AOT was the strongest unique predictor of support for democratic elections, and it uniquely predicted (low) support for censorship and discrimination as well. As might be expected, false polarization and political prejudice made unique, albeit weak, contributions to the political violence dimension, and false polarization also weakly predicted support for censorship. Additionally, utopianism and conspiracist ideation uniquely predicted lower support for democratic elections.

#### 4.1.4 Perceived System Legitimacy and Illegitimacy

Contrary to the hypotheses, perceived legitimacy and justification of the current system was generally not positively associated with antidemocratic attitudes (except support for censorship). Rather, the results suggest that perceived illegitimacy of the democratic system serves as a key mediator of the effects of ideological worldviews on antidemocratic attitudes in general. In other words, delegitimization of the government, the law, and the democratic institutions may play a key role not only in radicalization across different types of ideology [32] but also in transforming ideological worldviews—particularly system‐challenging sentiment (e.g., societal discontent)—into antidemocratic attitudes.

Nevertheless, it is important to keep in mind that this research focused specifically on the perceived illegitimacy of *the democratic system* and perceived legitimacy of *the government*. Interestingly, the belief that society is meritocratic was, contrary to our expectations, weakly positively associated with antidemocratic attitudes, suggesting that a general sense of societal legitimacy may play a role. However, it is also possible that the effects of perceived legitimacy of the government, specifically, are context‐dependent, emerging only in regimes that exhibit signs of autocracy or democratic decline. For example, some Americans may currently adopt more antidemocratic attitudes by virtue of legitimizing the Trump administration's policy agenda [3, 47].

### 4.2 Limitations and Future Directions

The most significant limitation of this study is the cross‐sectional nature of the design, which prevents causal inferences. In particular, a cross‐sectional comparison of predictors will privilege those that are conceptually or causally proximal to the outcome. This may, in this case, have been need for chaos, authoritarian aggression, and SDO, which encompass a desire to burn things to the ground and general support for violence and inequality, respectively. Future studies should use longitudinal studies to investigate whether changes in the predictors cause increases in antidemocratic attitudes and aim to develop a nuanced model of causal processes through which antidemocratic attitudes evolve over time. This includes, for example, examining the potential role of experiences of deprivation and unfairness as early antecedents [28, 29] and their potential interaction with need for chaos [4]. It includes examining the role of perceived illegitimacy of the democratic system and legitimacy of the government as mediators, particularly within regimes that exhibit ongoing democratic decline, and the role of epistemic attitudes in susceptibility to system‐delegitimizing misinformation or propaganda [9, 11, 32]. It also includes testing the potential moderating role of perceptions of threat to societal norms and ideological values in “activating” the antidemocratic potential of system‐justifying and system‐challenging worldviews [11, 48]. Furthermore, it would be worthwhile to examine the conditions under which the influence of ideological worldviews on antidemocratic tendencies can be reduced or redirected toward more normative political engagement. Previous studies highlight the potential role of psychological needs—particularly those stemming from political uncertainty, lack of control, and a disrupted sense of significance—in fostering support for autocratic leaders and radicalization [33, 36, 49].

Studies using multiwave designs along with statistical analyses that disentangle within‐ and between‐person change [50] and measures of attitudes to specific instances of democratic backsliding [47] would be particularly valuable. Causal effects in both directions need to be considered, as antidemocratic attitudes may, for example, make people more receptive to misinformation [51]. It is plausible that antidemocratic attitudes and some of the hypothesized predictors addressed in the current research mutually reinforce one another during the process of radicalization.

Furthermore, future studies might investigate whether interventions encouraging openness to new or alternative information and evidence may protect against antidemocratic attitudes, building on the literature on AOT in cognitive psychology [39]. The findings of the current study provide reason for hope, as epistemic attitudes are not set in stone. Past studies have found some effects of educational interventions designed to boost open‐minded thinking on myside‐bias and overconfidence [52, 53], but downstream effects on antidemocratic attitudes remain unexplored.

Although the measurement models were generally well‐supported, the limited homogeneity of the V‐dem measure of support for liberal democracy points to a need for further psychometric development and harmonization between the literatures on democracy in political science and psychology [11, 22]. The new four‐dimensional model of antidemocratic attitudes [11] is promising, but it is not necessarily comprehensive, and it is yet to be thoroughly scrutinized with respect to, for instance, invariance across populations and response tendencies. Similarly, the measurement of perceived system legitimacy and illegitimacy calls for further development. It may be possible to disentangle both legitimacy with respect to different aspects of the system, and different senses in which the system can be perceived as legitimate (or illegitimate)—for instance, the government may be seen as legitimate simply by virtue of being democratically elected or playing by democratic rules, or it may be seen as legitimate in a deeper ideological or moral sense, warranting unquestioned allegiance.

More research is also needed to replicate and refine the measurement models. The fact that predictors derived from disparate theoretical accounts in many cases correlated very strongly with one another illustrates the need for an integrative, cross‐disciplinary approach. At the same time, it is possible that some correlations were artificially inflated due to overlap between measures or response biases. Similarly, inattentive responses might inflate correlations when variables are skewed, insofar as they introduce random measurement error. Further psychometric scrutiny is thus warranted. It would be particularly worthwhile to develop cleaner, more distinct measures that better isolate specific aspects of ideological worldviews (e.g., by excluding system‐orthogonal elements from system‐justifying and system‐challenging predictors), along with hierarchical models that further integrate different conceptualizations.

Above all, it is important to test both measurement models and causal models across cultural contexts. The psychological literature on antidemocratic attitudes has hitherto been almost exclusively focused on the United States. Although this is an interesting case [3], democracy is also under threat in Hungary, Poland, Argentina, Turkey, the Philippines, India, Brazil, Venezuela, and many other countries worldwide [2]. Level of democracy and democratic backsliding are likely to play an important role in moderating associations between ideological worldviews and antidemocratic attitudes. For instance, in autocratic contexts, system‐justifying worldviews are particularly likely to be associated with a receptivity to autocratic propaganda and misinformation [23]. Conversely, system‐challenging worldviews are naturally less likely be manifested in antidemocratic attitudes when what they challenge is an autocratic system. To disentangle these kinds of moderating factors from other cultural and political factors, cross‐cultural data is needed.

## 5 Conclusion

Antidemocratic attitudes were associated both with ideological worldviews that justify and defend the current system and with those that challenge and undermine it, and these associations were mediated by perceived illegitimacy of the democratic system. They were also associated with characteristics that recur across ideological worldviews, including black‐and‐white thinking, a post‐truth mentality, a sense of being superior to others, and political prejudice and misperceptions. For the most part, these types of characteristics of ideological worldviews predicted antidemocratic attitudes independent of each other—this was true particularly for authoritarianism, SDO, need for chaos, prejudice against high‐status groups, lack of AOT, and misperceptions of antidemocratic attitudes among political opponents. These results show that an integrative account encompassing diverse predictors is needed to understand the psychology of antidemocratic tendencies.

## Author Contributions

A.N. conceptualized, designed, and conducted the research, analyzed the data, and wrote the paper. A.T. contributed to the design, conceptualization, and theoretical background.

## Conflicts of Interest

The authors declare that they have no competing interests.

## Supporting information



Supporting information about measures

Codebook

Data.csv

Supporting information

## Footnotes

^1^
https://osf.io/rjkd4

^2^
https://osf.io/ytuab
