# Supplementary material for: The Dark Side of Ideology: Ideological Worldviews and Antidemocratic Attitudes
Source: Ann N Y Acad Sci. 2025 Sep 27;1553(1):391–406. doi: 10.1111/nyas.70062 (PMC12645262; doi:10.1111/nyas.70062)
Supplement: Supplementary file 1 — Supporting information about measures [file NYAS-1553-391-s004.pdf]

Supporting information about structural equation modeling

This document contains supplemental information about analyses based on structural equation modeling. These analyses were run in AMOS 29.0 using Maximum Likelihood (ML) estimation. Model fit was evaluated in terms of the Root Mean Square Error of Approximation (RMSEA) with 90% confidence intervals and the Standardized Root Mean Squared Residual (SRMR). Common ideals are RMSEA < .06, and SRMR < .08 (Hu & Bentler, 1999). According to Kenny (2015), the Comparative Fit Index (CFI) compared to standardized fit criteria will be misleading and should not be reported when RMSEA of the independence model is lower than .158 (as it is impossible for CFI to satisfy the criteria in these cases). Therefore, we do not report CFI for these models, as the independence model consistently had RMSEA lower than this threshold.

To prevent convergence issues and distorted estimates due to multicollinearity, we first ran a model that included only those predictors that had stood out as the most robust and unique predictors in earlier regression analyses. The outcome was the full antidemocratic index. Items were used as indicators.

Thereafter, we ran a series of analyses to explore whether additional insight about the predictors and their robustness could be generated through tests of more comprehensive models. In a first step, we focused solely on the system justifying and system challenging worldviews, in a second, we focused on the system-orthogonal worldviews, and in the final step we included variables from both categories. In some cases, we used second-order factors to represent sets of predictors that were both closely theoretically related and very strongly correlated. In these models, items were used as indicators of the first-order factors for all predictors, but we used the four subscales (elections, censorship, discrimination, and violence) rather than individual items as indicators for antidemocratic attitudes as this turned out to reduce convergence issues and estimation errors.

All latent predictors were allowed to covary (when second-order factors were included, these were naturally the ones that were allowed to covary with other latent predictors).

Initial model with selected predictors

This initial model only included the predictors that have proved to be most robust in previous regression analyses, and the outcome was the full antidemocratic index (i.e., 31 items as indicators). Items were consistently used as indicators.

Model fit: $\chi^2(413) = 1567$ ($p < .001$), *SRMR* = .0674, *RMSEA* = .058[.056, .059] (R^2 = 66.1%; $\lambda \geq$ .24 for authoritarianism and .44 for all other scales; R^2 = 66.1%).

Standardized estimates are shown in the figure below.

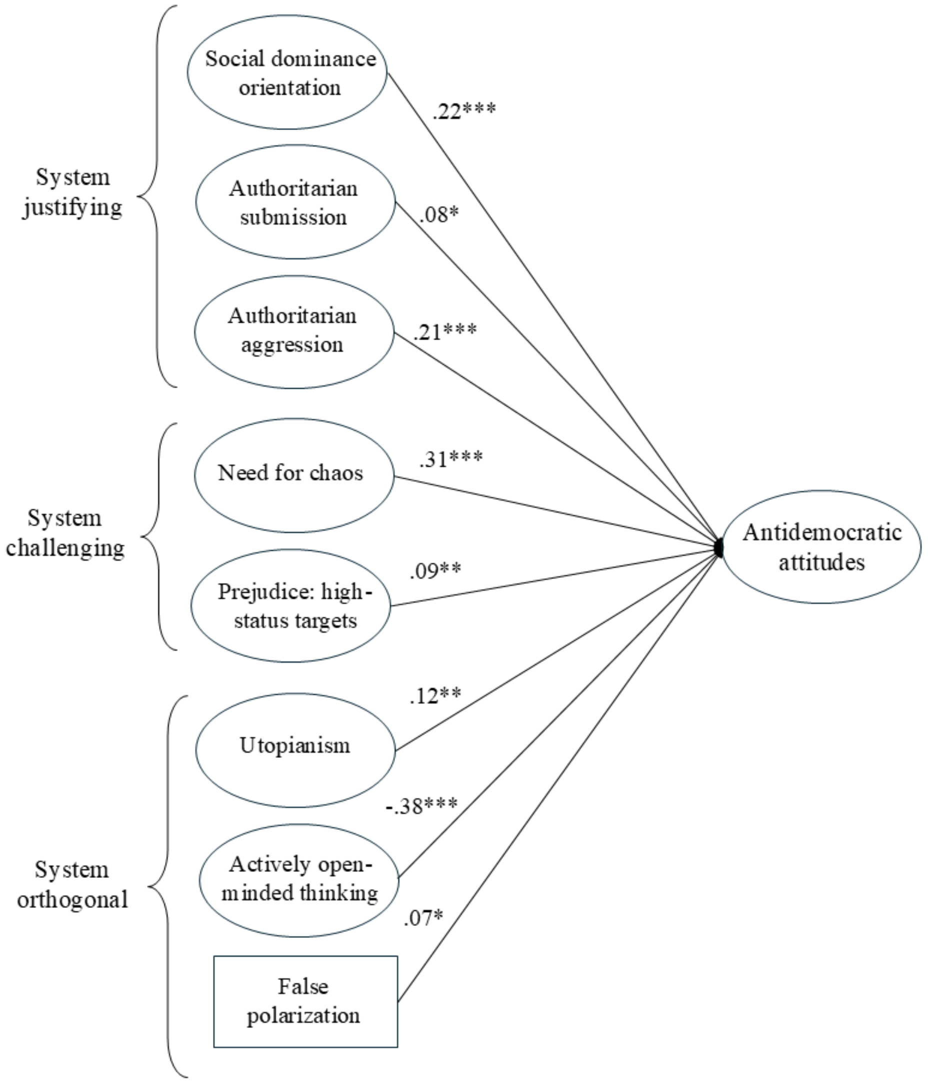

Supplementary Figure 1

Standardized path estimates. Predictors without significant effects in the hypothesized direction are omitted.

A follow-up model showed substantial variation in effects when the four subdimensions were used as outcomes. The democratic elections dimension (R^2 = 54.6%) was uniquely predicted by low AOT (γ = -.34), need for chaos (γ = .24), utopianism (γ = .18, p < 0.001), authoritarian aggression (γ = .14, p = .002), and SDO (γ = .12, p = .007). Censorship (R^2 = 40.3%) was uniquely predicted by authoritarian submission (γ = .25) and aggression (γ = .23, p < .001), low AOT (γ = -.20, p = .003), and false polarization (γ = .08, p = .023). Discrimination (R^2 = 66.8%) was uniquely predicted by SDO (γ = .44), authoritarian aggression (γ = .29), low AOT (γ = -.24, p < .001), and need for chaos (γ = .09, p = .094). Political violence (R^2 = 35.6%) was uniquely predicted by need for chaos (γ = .42), authoritarian aggression (γ = .22), prejudice against high-status targets (γ = .21, p < .001), and false polarization (γ = .09, p = .021).

System-justifying and system-challenging worldviews

Three second-order factors were initially included in this model:

(1) Authoritarianism: subsuming authoritarian aggression (λ = .56), authoritarian submission (λ = .77), and group-based authoritarianism (λ = .73)

(2) Societal malcontent: subsuming individual deprivation (λ = .94), group-based deprivation (λ = .86), perceived breakdown of society (λ = .65), and apocalypticism (λ = .50)

(3) Prejudice: subsuming prejudice against high-status targets (λ = .55) and prejudice against low-status targets (λ = .77)

For the third second-factor, we estimated paths from the first-order factors to antidemocratic attitudes, because we had separate hypotheses for the two types of prejudice. For the other two, we estimated paths from the second-order factors to antidemocratic attitudes.

The model also included separate first-order factors representing need for chaos, social dominance orientation, status-based risk-taking, and meritocratic world, as well as perceived system illegitimacy (including legitimacy items as reversed items) specified as a partial mediator of paths from all predictors to antidemocratic attitudes. Meritocratic world was not included underneath the societal malcontent factor, because it failed to load sufficiently strongly on this factor (λ = -.31). Distrust in politicians was excluded from the model because of a very strong correlation with the specified mediator (φ = .87).

Model fit: $\chi^2(2802) = 9513$ (p < .001), *SRMR* = .0865, *RMSEA* = .055[.053, .056]

Supplementary Table 1

Correlations between latent predictors in the model with second-order factors.

	1.	2.	3.	4.	5.	6.
1. Need for chaos						
2. Societal malcontent	.54					
3. Meritocratic world	-.25	-.25				
4. Prejudice	.68	.47	-.06			
5. Authoritarianism	.37	.14	.46	.43		
6. Social dominance	.29	.12	.07	.44	.50	
7. Status-driven risk taking	.60	.31	.10	.53	.45	.31

Supplementary Table 2.

Standardized estimates based on the mediation model with second-order factors.

	Total effect	Direct effect	Indirect effect
Need for chaos ($\lambda \geq .50$)	.24	.15	.10
Societal malcontent ($\lambda \geq .50$)	.14	-.07	.20
Meritocratic world ($\lambda \geq .75$)	.03	-.01	.04
Prejudice high-status targets ($\lambda \geq .65$)	.14	.03	.12
Authoritarianism ($\lambda \geq .63$)	.09	.66	-.27
Social dominance ($\lambda \geq .57$)	.45	.31	.15
Status-driven risk-taking ($\lambda \geq .47$)	-.08	-.08	.01
Prejudice low status targets ($\lambda \geq .74$)	-.11	-.07	-.04
System illegitimacy ($\lambda \geq .64$)	.45	.45	

The same model without second-order factors:

Model fit: $\chi^2(2669) = 8715$ ($p < .001$), *SRMR* = .0718, *RMSEA* = .053[.052, .054]

Supplementary Table 3

Correlations between latent predictors in the model without second-order factors.

	1.	2.	3.	4.	5.	6.	7.	8.	9.	10.	11.	12.
1. Need for chaos												
2. Breakdown of society	.44											
3. Individual deprivation	.51	.58										
4. Group-based deprivation	.43	.48	.85									
5. Apocalypticism	.31	.51	.42	.41								
6. Meritocratic world	-.10	-.15	-.25	-.20	-.19							
7. Prejudice: High status	.43	.43	.40	.36	.33	-.22						
8. Authoritarian submission	.21	-.05	-.03	-.05	-.19	.40	-.06					
9. Authoritarian aggression	.28	.30	.24	.13	.16	.12	.10	.27				
10. Group authoritarianism	.29	.21	.16	.14	.00	.37	.07	.60	.49			
11. Social dominance	.29	.27	.15	-.01	.00	.07	.08	.26	.50	.36		
12. Status-driven risk taking	.60	.29	.33	.20	.04	.10	.23	.31	.23	.38	.31	
13. Prejudice: Low status	.48	.26	.27	.16	.12	.04	.41	.26	.31	.31	.41	.42

Supplementary Table 5

Standardized estimates based on the mediation model without second-order factors.

	Total effect	Direct effect	Indirect effect
Need for chaos ($\lambda \geq .53$)	.28	.22	.06
Breakdown of society ($\lambda \geq .51$)	.03	-.06	.09
Individual deprivation ($\lambda \geq .55$)	.03	.03	.00
Group-based deprivation ($\lambda \geq .61$)	.02	-.01	.04
Apocalypticism ($\lambda \geq .65$)	.00	-.01	.01
Meritocratic world ($\lambda \geq .75$)	.11	.13	-.02
Prejudice high-status targets ($\lambda \geq .65$)	.16	.11	.05
Authoritarian submission ($\lambda \geq .33$)	.01	.17	-.16
Authoritarian aggression ($\lambda \geq .23$)	.27	.31	-.04
Group-based authoritarianism ($\lambda \geq .46$)	.12	.06	.05
Social dominance ($\lambda \geq .56$)	.47	.43	.04
Status-driven risk-taking ($\lambda \geq .47$)	-.03	-.02	-.01
Prejudice low status targets ($\lambda \geq .75$)	-.12	-.10	-.01
System illegitimacy ($\lambda \geq .65$)	.30	.30	

Next, we excluded the mediator and looked at variation in total effects on antidemocratic attitudes across different antidemocratic attitudes. We ran this analysis first with second-order factors, and thereafter without them (results without second-order factors in blue below).

Supplementary Table 6

Standardized estimates across different kinds of antidemocratic attitudes.

	Total	Elections	Censorship	Discrimination	Violence
Need for chaos	.24***	.19**	.08	.13*	.39***
Societal malcontent	.09*	.22***	-.09	.06	-.08
Breakdown of society	.01	.23***	-.13	-.08	-.06
Individual deprivation	.05	-.03	-.26*	.27*	-.23*
Group-based deprivation	.00	.13	.23*	-.24*	.20*
Apocalypticism	.01	-.08	.15*	.04	-.07
Meritocratic world	.01	.10*	-.09	.02	-.09
Prejudice: High status targets	.13***	.13***	.10*	.00	.25***
Authoritarianism	.41***	.28***	.60***	.26***	.13
Submission	.02	-.09	.36***	-.04	.07
Aggression	.29***	.04	.25***	.31***	.32***
Group-based	.10	.25***	-.02	.04	-.17*
Social dominance	.48***	.24***	.09	.72***	.02
Status-driven risk-taking	.09	.02	.14*	.13*	-.07
Prejudice: Low-status targets	-.12**	-.06	-.14**	-.08	-.07

Note. * $p < .05$, ** $p < .01$, *** $p < .001$

Summary

Authoritarianism, social dominance orientation, and need for chaos once again stand out as the best among these predictors, followed by societal malcontent and prejudice against high-status targets. There were, however, substantial variations across subdimensions of antidemocratic attitudes. Need for chaos, along with authoritarian aggression and prejudice against high-status groups, were clearly the strongest predictors of support for political violence, authoritarianism was by far the strongest predictor of support for censorship, and social dominance orientation was clearly the strongest predictor of the discrimination dimension, followed by authoritarianism. Furthermore, societal malcontent, including particularly perceived breakdown of the social fabric, stands out as a predictor specifically of willingness to restrict free elections. In other respects, none of the societal malcontent scales stand out as a particularly good unique predictor over and above effects of the others, and there are signs of statistical suppression when these scales are separated, with individual and group-based deprivation exhibiting effects in opposite directions in some cases. Authoritarian aggression was responsible for the largest part of the effect of authoritarianism, although submission and group-based authoritarianism also made some unique contributions.

Effects of the system-challenging predictors (particularly societal malcontent) as well as social dominance orientation were mediated by perceived illegitimacy of the system, whereas the effect particularly of authoritarian submission was mediated by perceived legitimacy of the system.

It is also notable that although there were for the most part stronger correlations within the categories of system-justifying and system-challenging worldviews, there were also some substantial correlations across the two categories. Most notably, need for chaos, and to a lesser extent perceived breakdown of the social fabric and relative deprivation, correlated *positively* with authoritarianism, social dominance, and status-driven risk-taking. These results suggest that dark varieties of system-justifying and system-challenging worldviews share some common characteristics.

System-orthogonal worldviews

Two second-order factors were included in this model:

(1) Narcissism: subsuming grandiosity (λ = .89), collective narcissism (λ = .79), and intellectual overconfidence (λ = .97)

(2) Simplistic epistemology: subsuming actively open-minded thinking (AOT; λ = -.84), conspiracist ideation (λ = .70), subjectivism (λ = .63), dichotomous epistemology (λ = .81), utopianism (λ = .66), Manicheanism (λ = .65), and distrust in experts (λ = .69)

The reason that post-truth attitudes and black-and-white thinking were not represented as separate second-order factors is that this would lead to estimation issues, as there was a lot of overlap between these factors (see Supplementary Table 8 below).

Model with second-order factors:

Model fit: $\chi^2(1576) = 5170$ ($p < .001$), *SRMR* = .0670, *RMSEA* = .053[.052, .055]

Supplementary Table 7

Correlations between predictors in the model with second-order factors.

	1.	2.	3.
1. Narcissism			
2. Simplistic epistemology	.66		
3. Political prejudice	.31	.30	
4. False polarization	.20	.07	.30

Model without second-order factors:

Model fit: $\chi^2(1518) = 4731$ ($p < .001$), *SRMR* = .0581, *RMSEA* = .051[.050, .053]

Supplementary Table 8

Correlations between predictors in the model without second-order factors.

	1.	2.	3.	4.	5.	6.	7.	8.	9.	10.	11.
1. Grandiosity											
2. Collective narcissism	.70										
3. Intellectual overconfidence	.88	.73									
4. Conspiracist ideation	.27	.46	.38								
5. Subjectivism	.33	.36	.32	.46							
6. AOT	-.50	-.47	-.61	-.47	-.59						
7. Dichotomous epistemology	.54	.48	.60	.49	.45	.72					
8. Utopianism	.37	.44	.46	.47	.40	.57	.58				
9. Manicheanism	.26	.45	.36	.62	.41	.50	.48	.48			
10. Distrust in experts	.33	.40	.48	.61	.47	.56	.56	.28	.45		
11. Political prejudice	.27	.36	.26	.30	.13	.22	.19	.24	.22	.24	
12. False polarization	.15	.24	.19	.12	.03	.00	.02	.12	.03	.06	.30

Supplementary Table 9

Standardized estimates across different kinds of antidemocratic attitudes (estimates based on the model without second-order factors are marked in blue)

	Total	Elections	Censorship	Discrimination	Violence
Narcissism ($\lambda \geq .79$)	.09	-.04	.07	.27***	.19***
Grandiosity ($\lambda \geq .69$)	.13	.05	.34*	-.04	.33*
Collective narcissism ($\lambda \geq .59$)	-.09	-.05	-.01	-.14	-.08
Intellectual overconfidence ($\lambda \geq .56$)	.04	.02	-.35	.35*	-.11
Simplistic epistemology ($\lambda \geq .65$)	.71***	.74***	.37***	.37***	.14
Conspiracist ideation ($\lambda \geq .73$)	.24***	.28***	-.10	.01	.31***
Subjectivism ($\lambda \geq .71$)	-.01	.04	-.07	.01	-.14*
AOT ($\lambda \geq .40$)	-.38***	-.32***	-.35***	-.32**	-.06
Dichotomous epistemology ($\lambda \geq .62$)	.15*	.06	.11	.31***	.10
Utopianism ($\lambda \geq .67$)	.13*	.19***	.10	-.13*	.08
Manicheanism ($\lambda \geq .71$)	-.14**	-.13**	.00	-.05	-.19***
Distrust in experts ($\lambda \geq .55$)	.11	.12	-.05	.11	-.09
Political prejudice ($\lambda \geq .72$)	-.03	-.03	-.03	-.18***	.25***
False polarization	.12**	.09*	.09	.03	.13**

Note. * $p < .05$, ** $p < .01$, *** $p < .001$

Summary

The scales measuring black-and-white thinking and post truth-mentality were strongly correlated and could be modeled with a common secondary factor (i.e., simplistic epistemology), which in turn correlated very strongly with a factor representing perceived superiority of the self and ingroup (i.e., general narcissism). These results suggest that there is indeed a cohesive personality pattern across these different predictors. Nevertheless, the simplistic epistemology factor was by far the better predictor of antidemocratic attitudes, strongly predicting all subdimensions except support for political violence. Most of this effect was explained by low actively open-minded thinking, although conspiracist ideation also had unique effects on the total, elections, and violence dimensions, and there were some weaker effects of dichotomous epistemology and utopianism as well. There were negative effects of Manicheanism, indicating that there was statistical suppression when the epistemological predictors were modeled without a common second-order factor.

Narcissism had some effects on support for censorship and political violence. Grandiosity was the best of the narcissism predictors, and there were signs of suppression effects when grandiosity and intellectual overconfidence were included as separate predictors. False polarization was overall a better predictor of antidemocratic attitudes than political prejudice, although political prejudice made a substantial contribution to the prediction of support for political violence.

Integrated model: system-justifying, -challenging, and -orthogonal worldviews

Drawing on the previous results, these analyses tested a series of models using predictors representing all types of ideological worldviews.

1. The first model included all second-order factors (narcissism, simplistic epistemology, authoritarianism, societal malcontent, and prejudice), as well as need for chaos, social dominance, and risk-taking as separate first-order factors, and false polarization as a manifest predictor.
    a. Version 1: The outcome was originally a latent variable with the four antidemocratic attitude scales as indicators.
    b. Versions 2-5: The model was run four additional times with each of the four antidemocratic attitude dimensions as outcomes and items as indicators
    c. Version 6: The model was run with all four antidemocratic attitude scales as manifest outcomes in the same model.
2. The second model was the most comprehensive model with all predictors as separate first-order factors. The outcome was a latent variable with the four antidemocratic attitude scales as indicators. Rather than testing additional versions of this model, we proceeded to exclude some predictors that had consistently failed to show any unique positive effects at this point.
3. The third model retained narcissism, societal malcontent, and prejudice as second-order factors, but substituted the authoritarianism factor for separate submission and aggression factors, and the simplistic epistemology factor for separate AOT, conspiracist ideation, simplistic epistemology, and utopianism factors. In other respects, the procedure was the same as for the first model above:
    a. Version 1: The outcome was originally a latent variable with the four antidemocratic attitude scales as indicators.
    b. Versions 2-5: The model was run four additional times with each of the four antidemocratic attitude dimensions as outcomes and items as indicators
    c. Version 6: The model was run with all four antidemocratic attitude scales as manifest outcomes in the same model.

Model with all second-order factors:

Model fit: $\chi^2(6489) = 17713$ ($p < .001$), *SRMR* = .0806, *RMSEA* = .046[.046, .047] (R^2 = 81.6%)

Supplementary Table 10

Correlations between predictors in the model with second-order factors.

	1.	2.	3.	4.	5.	6.	7.	8.
1. Need for chaos								
2. Societal malcontent	.60							
3. Authoritarianism	.37	.21						
4. Social dominance	.30	.18	.51					
5. Status-driven risk-taking	.60	.31	.44	.31				
6. Narcissism	.70	.45	.58	.49	.63			
7. Simplistic epistemology	.56	.72	.60	.46	.46	.64		
8. Prejudice	.50	.59	.14	.16	.32	.39	.64	
9. False polarization	.25	.27	.01	.07	.10	.20	.08	.30

Supplementary Table 11

Standardized estimates across different kinds of antidemocratic attitudes

	Total	Elections	Censorship	Discrimination	Violence
Need for chaos	.27***	.20**	.13	.11	.46***
Societal malcontent	-.15	-.09	-.14	-.15	-.05
Prejudice: High status	.15**	.11*	.09	.07	.21***
Authoritarianism	.26***	.13*	.52***	.13	.16*
Social dominance	.39***	.13**	.11*	.65***	.08
Status-driven risk-taking	-.08	.00	-.13	-.15*	-.09
Prejudice: Low-status	-.03	.03	-.11*	-.03	-.06
Narcissism	-.16*	-.23***	-.08	.02	-.14
Simplistic epistemology	.49***	.68***	.04	.34***	-.13
Political prejudice	-.08	-.11*	.04	-.13*	.10
False polarization	.12**	.10*	.11*	.06	.09*

Note. * $p < .05$, ** $p < .01$, *** $p < .001$

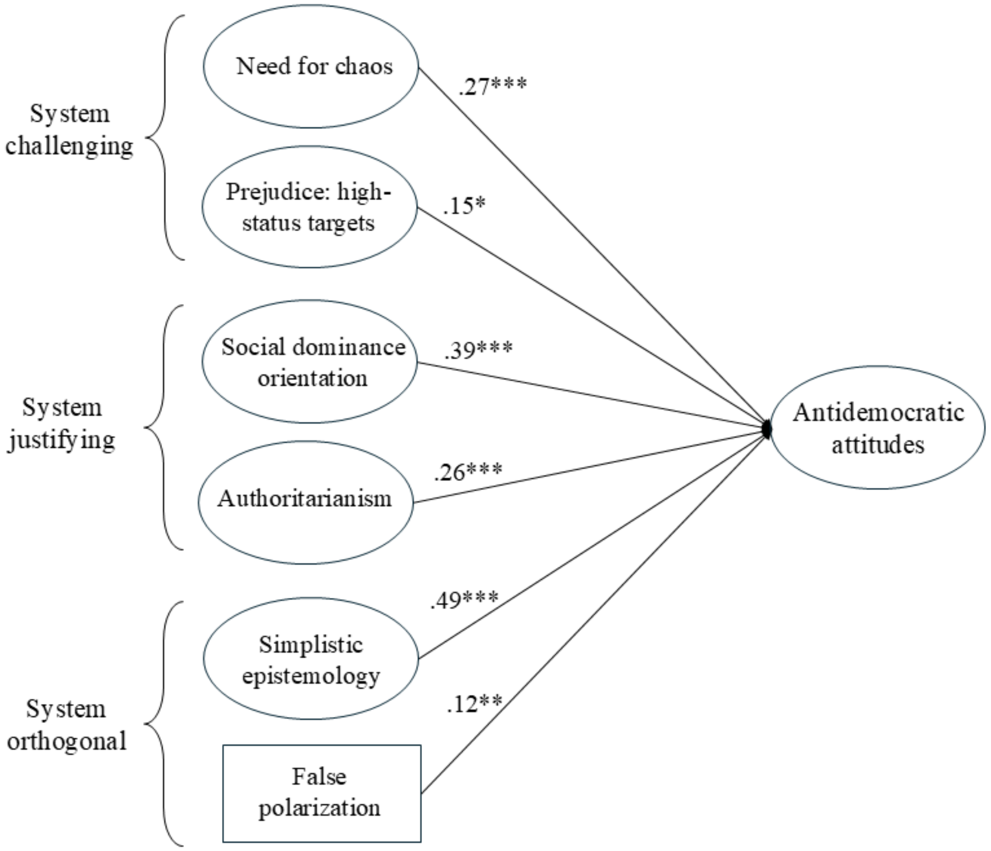

Supplementary Figure 2

Standardized effects on total antidemocratic attitudes. Predictors without significant effects in the hypothesized direction are omitted.

Supplementary Table 12

Standardized estimates with the four manifest antidemocratic outcome variables

	Elections<br>R^2 = 49.1%	Censorship<br>R^2 = 24.8%	Discrimination<br>R^2 = 51.1%	Violence<br>R^2 = 33.8%
Need for chaos	.19**	.11	.10	.44***
Societal malcontent	-.06	-.11	-.14	-.05
Prejudice: High status	.10*	.08	.06	.19***
Authoritarianism	.13*	.46***	.12*	.17*
Social dominance	.11**	.10*	.52***	.07
Status-driven risk-taking	-.01	-.11	-.11*	.08
Prejudice: Low-status	.03	-.09*	-.03	-.06
Narcissism	-.22***	-.08	.00	-.15*
Simplistic epistemology	.60***	.03	.28**	-.10
Political prejudice	-.10*	.04	-.09*	.09*
False polarization	.09*	.09*	.06	.09*

Note. * $p < .05$, ** $p < .01$, *** $p < .001$

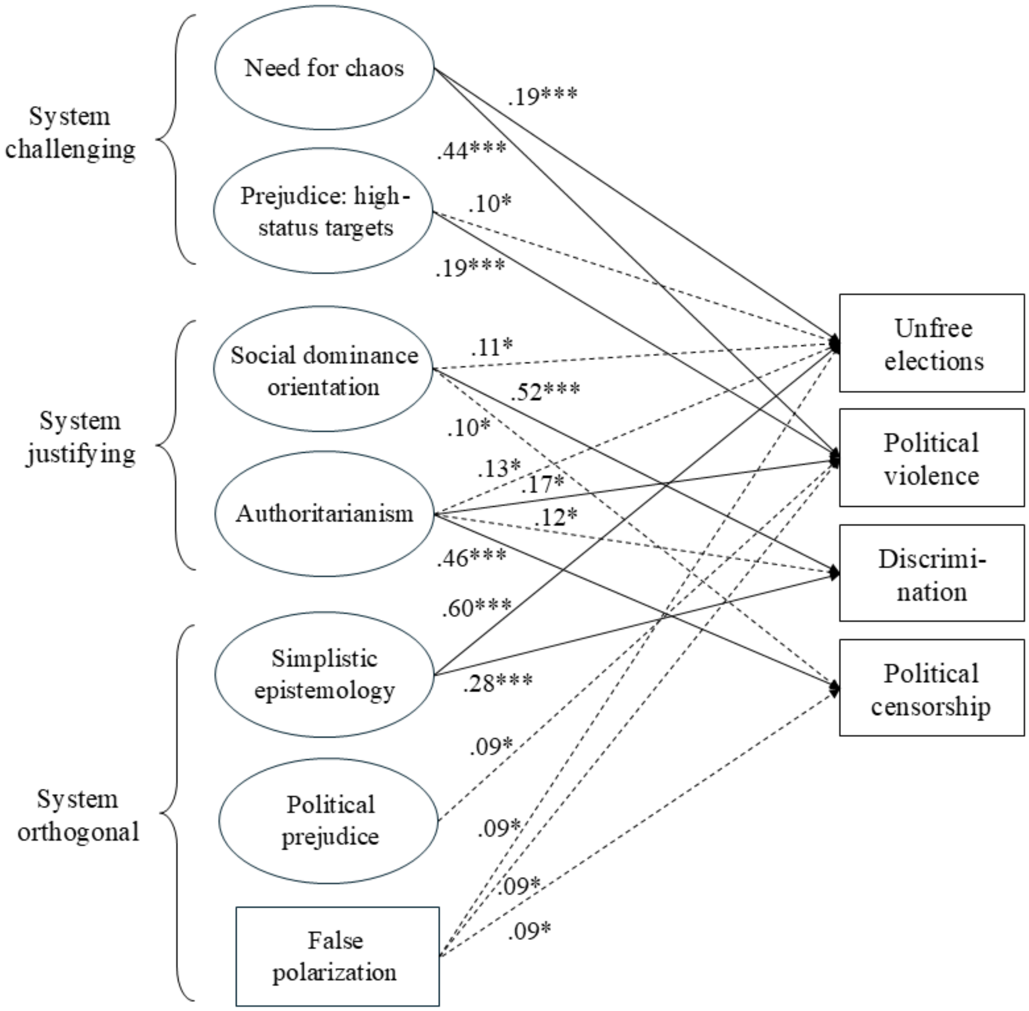

Supplementary Figure 3

Standardized effects on total antidemocratic attitudes. Predictors without significant effects in the hypothesized direction and non-significant paths are omitted.

Model without second-order factors:

Model fit: $\chi^2(5440) = 14294$ ($p < .001$), *SRMR* = .0608, *RMSEA* = .045[.044, .046] ($R^2 = 85.6\%$)

Supplementary Table 13

Standardized estimates of effects on general antidemocratic attitudes

	Antidemocratic attitudes
Need for chaos	.21**
Breakdown of society	-.09
Relative deprivation	-.03
Apocalypticism	.02
Prejudice: High status targets	.16**
Authoritarian submission	.08
Authoritarian aggression	.34***
Social dominance	.36***
Status-driven risk-taking	.03
Prejudice: Low-status targets	-.06
Grandiosity	-.14
Collective narcissism	.01
Conspiracist ideation	.17**
Subjectivism	.07
AOT	-.32***
Dichotomous epistemology	.04
Utopianism	.08
Manicheanism	-.16**
Distrust in experts	.00
Political prejudice	-.08
False polarization	.07

Note. * $p < .05$, ** $p < .01$, *** $p < .001$

Supplementary Table 14

Correlations between predictors in the model without second-order factors.

	1.	2.	3.	4.	5.	6.	7.	8.	9.	10.	11.	12.	13.	14.	15.	16.	17.	18.	19.	20.
1. Need for chaos																				
2. Breakdown of society	.44																			
3. Relative deprivation	.49	.55																		
4. Apocalypticism	.31	.51	.44																	
5. Prejudice: High status	.43	.42	.40	.12																
6. Authoritarian submission	.22	-.04	-.03	-.19	-.06															
7. Authoritarian aggression	.28	.30	.18	.16	.10	.26														
8. Social dominance	.30	.27	.07	.00	.08	.26	.50													
9. Status-driven risk-taking	.60	.28	.27	-.04	.23	.31	.23	.31												
10. Prejudice: Low-status	.49	.26	.22	.12	.41	.26	.31	.41	.42											
11. Grandiosity	.65	.27	.29	.05	.22	.40	.40	.48	.63	.56										
12. Collective narcissism	.60	.39	.68	.27	.33	.19	.32	.24	.45	.39	.69									
13. Conspiracist ideation	.48	.60	.52	.38	.40	-.08	.23	.23	.31	.26	.26	.46								
14. Subjectivism	.35	.42	.35	.15	.20	.11	.17	.18	.29	.19	.33	.35	.46							
15. AOT	-.42	-.41	-.34	-.10	-.24	-.31	-.31	-.50	-.41	-.33	-.50	-.46	-.46	-.59						
16. Dichotomous epistemology	.46	.46	.36	.11	.22	.34	.43	.50	.45	.35	.54	.48	.49	.45	-.72					
17. Utopianism	.40	.39	.27	.20	.25	.29	.29	.21	.40	.29	.37	.44	.47	.40	-.57	.58				
18. Manicheanism	.27	.48	.36	.32	.31	.12	.29	.20	.21	.18	.26	.45	.62	.41	-.50	.48	.48			
19. Distrust in experts	.41	.51	.44	.18	.30	-.07	.31	.43	.25	.28	.33	.39	.61	.47	-.55	.56	.28	.45		
20. Political prejudice	.36	.36	.36	.25	.74	-.02	.11	.12	.23	.43	.27	.36	.30	.13	-.22	.19	.24	.22	.24	
21. False polarization	.24	.24	.16	.19	.21	-.01	.11	.06	.09	.13	.15	.24	.12	.03	.01	.01	.12	.04	.06	.30

Revised model without some second-order factors:

Model fit: $\chi^2(4749) = 13874$ ($p < .001$), *SRMR* = .0796, *RMSEA* = .049[.048, .050] (R^2 = 84.6%)

Supplementary Table 15

Standardized estimates across different kinds of antidemocratic attitudes

	Total	Elections	Censorship	Discrimination	Violence
Need for chaos	.28***	.17*	.12	.14	.50***
Societal malcontent	-.15	.05	-.15	-.18	-.27**
Prejudice: High status	.15**	.11*	.09	.08	.21***
Authoritarian submission	.03	.05	.28***	-.07	-.01
Authoritarian aggression	.34***	.13*	.27***	.35***	.28***
Social dominance	.33***	.14**	.07	.52***	-.03
Status-driven risk-taking	.04	.02	-.09	-.10	.10
Prejudice: Low-status	-.04	.03	-.11**	-.03	-.06
Narcissism	-.16*	-.18*	-.10	-.02	-.20**
Conspiracist ideation	.14*	.21***	-.01	.06	.08
AOT	-.34***	-.35***	-.25**	-.25**	-.01
Dichotomous epistemology	.03	-.03	-.04	.13	.04
Utopianism	.07	.15**	.05	-.05	.01
Political prejudice	-.09	-.13**	.03	-.12*	.12*
False polarization	.08*	.05	.09	.04	.09*

Note. * $p < .05$, ** $p < .01$, *** $p < .001$

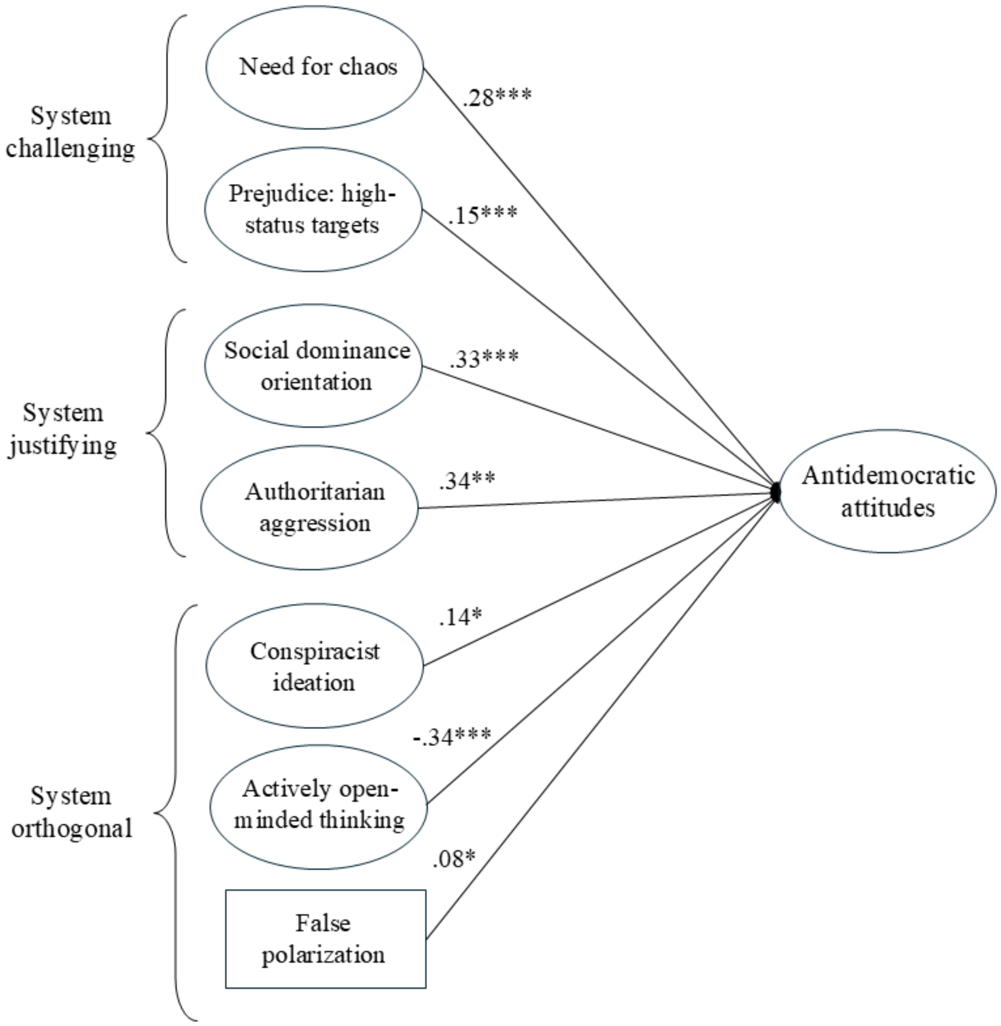

Supplementary Figure 4

Standardized effects on total antidemocratic attitudes. Predictors without significant effects in the hypothesized direction are omitted.

Model fit: $\chi^2(5059) = 14674$ ($p < 0.001$), *SRMR* = .0796, *RMSEA* = .049[.048, .050] ($R^2 = 81.6\%$)

Supplementary Table 16

Standardized estimates across different kinds of antidemocratic attitudes in a model with authoritarianism represented by a second-order factor

	Total
Need for chaos	.25***
Societal malcontent	-.01
Prejudice: High status	.15**
Authoritarianism	.33***
Social dominance	.39***
Status-driven risk-taking	-.07
Prejudice: Low-status	-.04
Narcissism	-.14*
Conspiracist ideation	.14*
AOT	-.26***
Dichotomous epistemology	-.03
Utopianism	.05
Political prejudice	-.10*
False polarization	.10**

Note. * $p < .05$, ** $p < .01$, *** $p < .001$

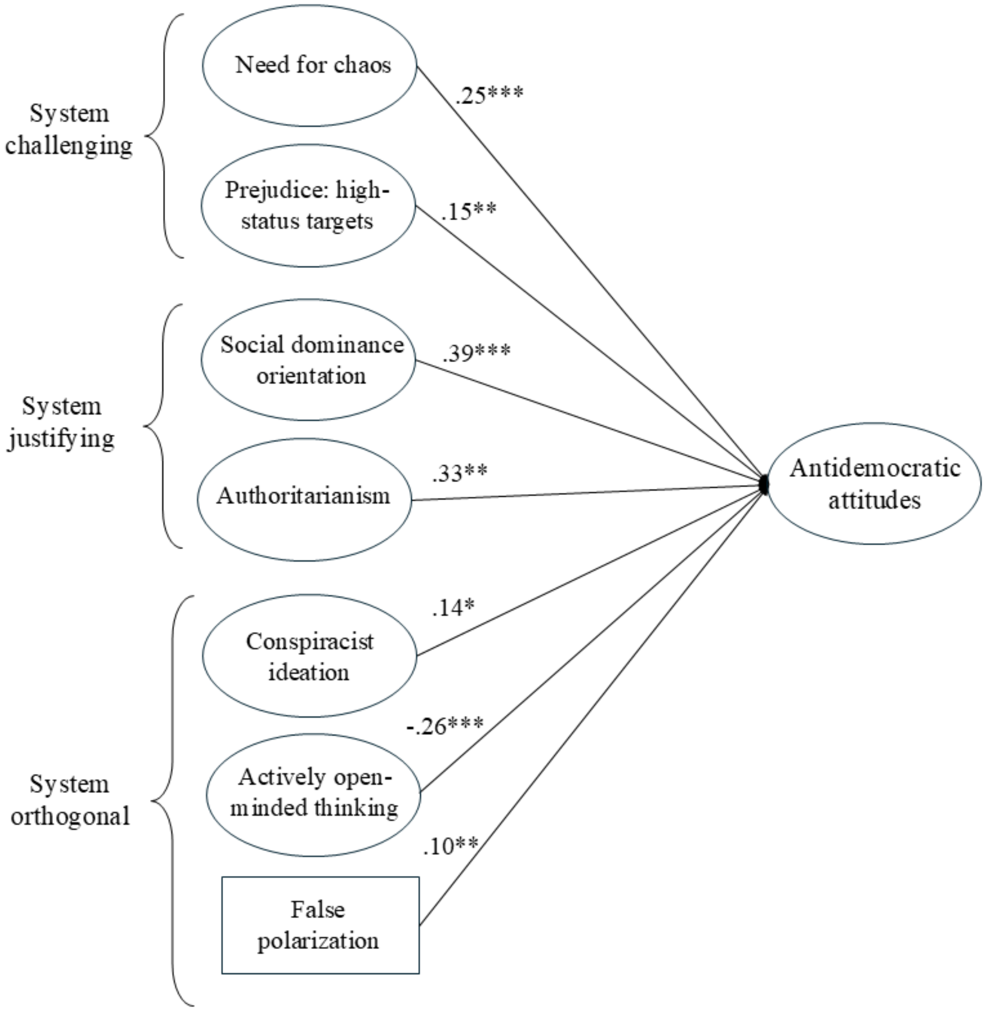
System challenging
Need for chaos
.25***
Prejudice: high-status targets
.15**
System justifying
Social dominance orientation
.39***
Authoritarianism
.33**
System orthogonal
Conspiracist ideation
.14*
Actively open-minded thinking
-.26***
False polarization
.10**
Antidemocratic attitudes

Supplementary Table 17

Standardized estimates with the four manifest antidemocratic outcome variables

	Elections R^2 = 47.4%	Censorship R^2 = 25.7%	Discrimination R^2 = 54.9%	Violence R^2 = 37.4%
Need for chaos	.16*	.10	.12*	.48***
Societal malcontent	.06	-.12	-.17*	-.26**
Prejudice: High status	.10*	.08	.07	.19***
Authoritarian submission	.04	.25***	-.06	-.02
Authoritarian aggression	.11*	.24***	.29***	.26***
Social dominance	.13**	.07	.41***	-.02
Status-driven risk-taking	.01	-.08	-.08	.09
Prejudice: Low-status	.03	-.10**	-.03	-.05
Narcissism	-.16*	-.10	-.02	-.19**
Conspiracist ideation	.19***	-.01	.05	.09
AOT	-.31***	-.22**	-.22***	-.02
Dichotomous epistemology	-.02	-.03	.08	.04
Utopianism	.14**	.04	-.03	.01
Political prejudice	-.12**	.03	-.09*	.11*
False polarization	.05	.08	.04	.09*

Note. * $p < .05$, ** $p < .01$, *** $p < .001$

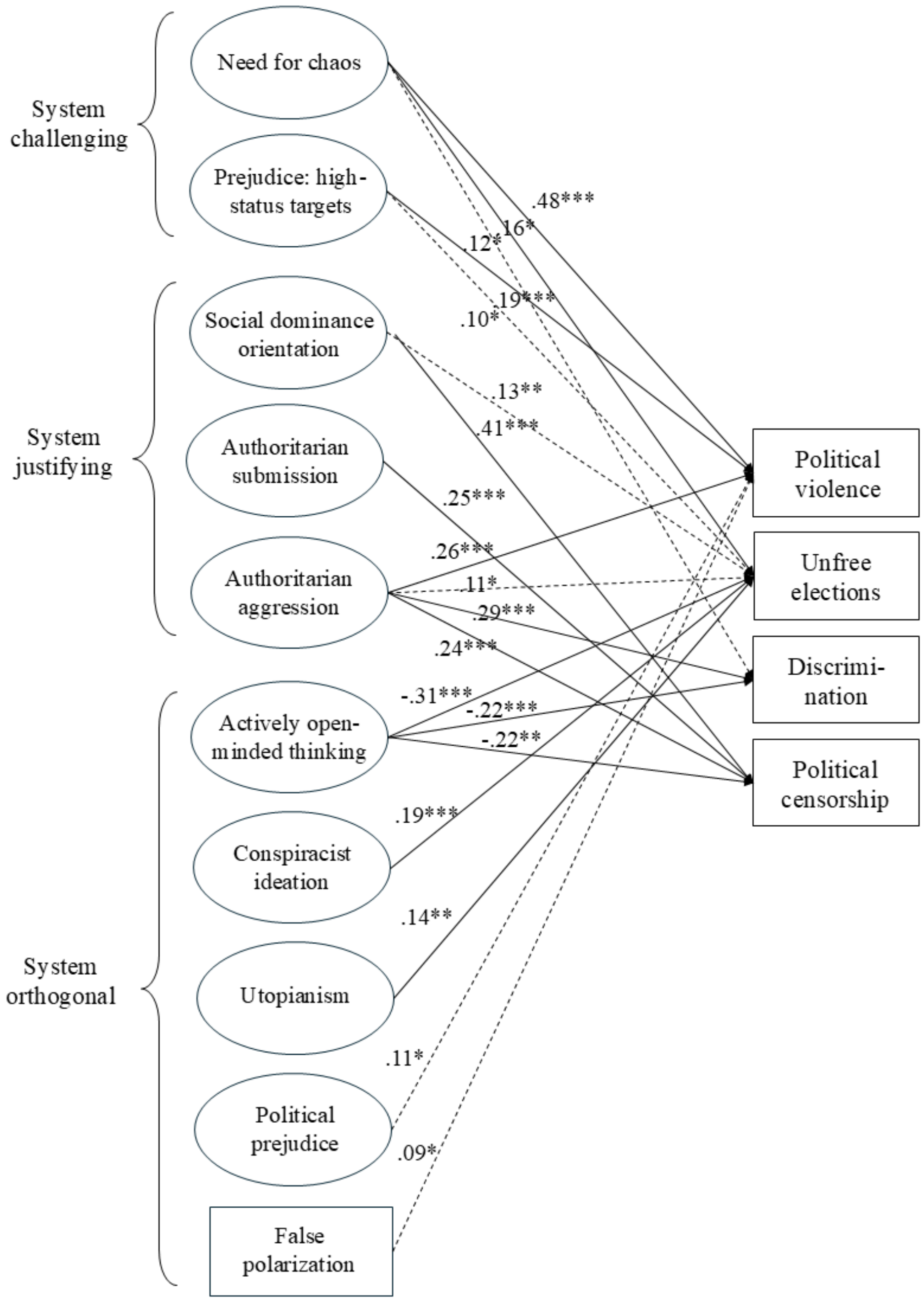

Supplementary Figure 5

Standardized effects on total antidemocratic attitudes. Predictors without significant effects in the hypothesized direction and non-significant paths are omitted.

Supplementary Table 18

Standardized estimates with the four manifest antidemocratic outcome variables in a model with authoritarianism represented by a second-order factor

	Elections<br>R^2 = 48.4%	Censorship<br>R^2 = 26.3%	Discrimination<br>R^2 = 50.6%	Violence<br>R^2 = 33.8%
Need for chaos	.15*	.11	.08	.44***
Societal malcontent	.11	-.06	-.05	-.16
Prejudice: High status	.10*	.08	.07	.18***
Authoritarianism	.22**	.49***	.15*	.12
Social dominance	.14***	.09	.51***	.06
Status-driven risk-taking	-.01	-.11	-.11*	.06
Prejudice: Low-status	.02	-.09*	-.03	-.05
Narcissism	-.17**	-.10	.01	-.15*
Conspiracist ideation	.19***	-.04	.05	.09
AOT	-.28***	-.17*	-.14*	.05
Dichotomous epistemology	-.08	-.12	.05	.03
Utopianism	.11*	-.01	-.04	.01
Political prejudice	-.12**	.02	-.10*	.10*
False polarization	.06	.10*	.05	.10**

Note. * $p < .05$, ** $p < .01$, *** $p < .001$

Summary

System justifying and system challenging worldviews

Tests of the integrated model confirmed that authoritarianism, social dominance orientation, need for chaos, and prejudice against high-status targets were the most robust system-justifying and system-challenging predictors of antidemocratic attitudes. These analyses made it even clearer that need for chaos, authoritarian aggression, and prejudice against high-status groups were the best unique predictors of support for political violence, while authoritarianism was by far the best predictor of support for censorship, and social dominance orientation was by far the best predictor of the discrimination dimension. Authoritarian aggression was the most consistent predictor of different kinds of antidemocratic attitudes overall, while authoritarian submission more specifically predicted support for censorship, and it was the only one of these predictors that was associated with higher rather than lower perceived legitimacy of the system. The effects of social dominance orientation and particularly authoritarianism were reduced by overlap with some of the system-orthogonal predictors.

Although earlier analyses suggested that there was a unique effect of societal malcontent, including particularly, societal perceived breakdown of the social order, on general antidemocratic attitudes mediated by perceived illegitimacy of the system, effects of this factor vanished or reversed (due to statistical suppression) in the full model. This is because perceived breakdown of the social fabric and relative deprivation were extremely strongly associated with other factors, including simplistic epistemology (particularly conspiracist ideation), prejudice, need for chaos, and collective narcissism (in the case of relative deprivation).

System orthogonal worldviews

The simplistic epistemology factor, covering both black-and-white thinking and post-truth-mentality was, once again, a very strong and robust predictor of all types of antidemocratic attitudes except support for political violence. Actively open-minded thinking accounted for most of this effect, and it had very strong correlations with all of the other black-and-white and post-truth mentality factors and had the highest (negative) loading on the second order factor. Nevertheless, conspiracist ideation and utopianism also made unique contributions to the prediction of willingness to sacrifice free elections. Furthermore, false polarization also made a marginal unique contribution to the prediction of general antidemocratic attitudes, and political prejudice marginally contributed to the prediction of support for political violence.

Similar to societal malcontent, perceived superiority of the self and ingroup made no unique contribution to the prediction of antidemocratic attitudes. The effects all vanished or reversed due to overlap with other predictors. The second-order narcissism factor was indeed very strongly correlated with need for chaos, authoritarianism, and simplistic epistemology. Collective narcissism was extremely strongly correlated with relative deprivation, while grandiosity was particularly strongly correlated with status-driven risk taking and prejudice against low-status groups.
