# Supplementary material for: The Dark Side of Ideology: Ideological Worldviews and Antidemocratic Attitudes
Source: Ann N Y Acad Sci. 2025 Sep 27;1553(1):391–406. doi: 10.1111/nyas.70062 (PMC12645262; doi:10.1111/nyas.70062)
Supplement: Supplementary file 2 — Codebook [file NYAS-1553-391-s003.pdf]

Codebook

Reversed item *

Metadata

duration: Duration of participation in seconds

recaptcha: the Qualtrics “Q_RecaptchaScore” which can be used to identify bots

fraud: the Qualtrics “Q_RelevantIDFraudScore” which can be used to identify bots and fraudulent responses

exclude: filter to identify participants who failed to satisfy inclusion criteria (0 = do not exclude, 1 = exclude)

Antidemocratic attitudes

antidemo1: “People should be free to criticize the government even in times of great crisis” *

antidemo2: “The government should be able to censor media sources that are too critical”

antidemo3: “This country would be better off if there were only one political party”

antidemo4: “The right to protest should be protected even when protestors inconvenience others” *

antidemo5: “The government should have the power to ban organizations that promote subversive values”

antidemo6: “The universal right to vote must be questioned when so many voters are poorly informed and easily misled”

antidemo7: “All adult British citizens should have the right to vote, even individuals holding extreme views” *

antidemo8: “Our government would run better if decisions were left up to non-elected independent experts rather than politicians or the people”

antidemo9: “Non-political authorities, such as the National Health Service (NHS), should never be able to overrule elected politicians” *

antidemo10: “We should respect the results of elections, no matter which party wins” *

antidemo11: “Governments are justified in bending electoral rules in their favor when their opponents have also done so in the past”

antidemo12: “British courts should be able to overrule the government if policies are judged to be illegal” *

antidemo13: “The government should be able to ignore court rulings that are regarded as politically biased”

antidemo14: “If Parliament hinders the work of the government, it should be ignored”

antidemo15: “Members of Parliament should be able to question and oversee political decisions taken by the government, even when this slows down progress” *

antidemo16: “The government should be able to bend the law to solve pressing social and political problems”

antidemo17: “All British people should enjoy the same legal rights, regardless of their political beliefs” *

antidemo18: “Having a strong leader who is not elected is sometimes better than democracy”

antidemo19: “A political system without parties and elections can be preferable when people vote for the wrong politicians”

antidemo20: “Whether we live in a democracy or under a dictatorship makes no difference”

antidemo21: “Since elections sometimes produce bad results, we should adopt other methods for choosing our leaders”

antidemo22: “It is more important to have a government that can get things done than living in a democracy”

antidemo23: “Being a UK citizen should not be enough to get the same rights as everyone else”

antidemo24: “Minorities have no right to make demands on the majority in society”

antidemo25: “Sometimes it is right to sacrifice the rights of some individuals or groups for the best of society”

antidemo26: “It is more important that the laws protect our country’s interests than that all individuals have the same right to one hundred percent”

antidemo27: “Political messages from some groups should be blocked or censored from the public”

antidemo28: “Today, people have too much freedom of speech in our country”

antidemo29: “Silencing critical voices and limiting political opposition is sometimes necessary for the good of the country”

antidemo30: “Newspapers should censor authors who express extreme political opinions”

antidemo31: “Some groups should not be allowed to make a speech or demonstrate in public places”

antidemo32: “Even in a democracy, using violence to reach your political goals can be justified under some circumstances”

antidemo33: “In certain situations, there is no option but to use arms to prevent the government from implementing its policies”

antidemo34: “attacking your political opponents can be acceptable under some circumstances”

antidemo35: “When national disaster is near and all other forms of civil protest have failed, physically injuring politicians is permissible”

antidemo36: “Political violence can be constructive when it serves the right cause”

Authoritarianism and social dominance orientation

sdo1: “An ideal society requires some groups to be on top and others to be on the bottom”

sdo2: “Some groups of people are simply inferior to other groups”

sdo3: “No one group should dominate in society” *

sdo4: “Groups at the bottom are just as deserving as groups at the top” *

sdo5: “Group equality should not be our primary goal”

sdo6: “It is unjust to try to make groups equal”

sdo7: “We should do what we can to equalize conditions for different groups” *

sdo8: “We should work to give all groups an equal chance to succeed” *

submission1: “We should believe what our leaders tell us”

submission2: “Our leaders know what is best for us”

submission3: “Questioning the motives of those in power is healthy for society” *

submission4: “People should never question statements made by those in positions of authority”

aggression1: “Strong force is necessary against threatening groups”

aggression2: “It is necessary to use force against people who are a threat to authority”

aggression3: “People should avoid using violence against others even when ordered to do so by the proper authorities” *

aggression4: “Strong punishments are necessary in order to send a message”

groupauth1: “A group member should always obey group rules”

groupauth2: “A group member who has violated group rules should be punished severely”

groupauth3: “If a group has a leader, group members have to respect and obey him/her under all circumstances”

attcheck1: “Please respond "Disagree" in this row to show that you are paying attention”

——————— Breakdown of social fabric, cynicism, dangerous-world belief, apocalypticism, meritocratic world, group-based deprivation, individual deprivation ———————

breakdown1: “People think that there are no clear moral standards to follow”

breakdown2: “Most people think that if something works, it doesn’t really matter whether it is right or wrong”

breakdown3: “Most people think that honesty doesn’t work all the time; dishonesty is sometimes a better approach to get ahead”

breakdown4: “People do not know who they can trust and rely on”

cynicism1: “In today’s world many people take advantage of an unselfish person”

cynicism2: “People pretend to care more about one another than they really do”

cynicism3: “Human beings tend to exploit others”

cynicism4: “Most people would tell a lie if they could benefit from it”

dang1: “Basically, this world is generally a dangerous place”

dang2: “We do not live in a dangerous world” *

dang3: “Society today is lawless and bestial”

dang4: “The world is pretty safe” *

apoc1: “Drastic action is necessary to save the world from its problems”

apoc2: “Civilization will come to an end unless we make radical changes”

apoc3: “The world is headed for destruction”

apoc4: “Today the human race is on the edge of an enormous calamity”

merit1: “A person's efforts are usually noticed and rewarded”

merit2: “People who do their job well rise to the top”

merit3: “People who excel are generally acknowledged and rewarded”

merit4: “In general, people are rewarded based on their merits and achievements”

groupdep1: “I think that people in my group are disadvantaged because society oppresses them”

groupdep2: "I feel furious about the limited opportunities of people in my group to get ahead in their lives"

groupdep3: "I feel angry because society discriminates against people in my group"

groupdep4: "My group will always be at the bottom and others at the top of the social ladder"

inddep1: "When compared to others, people like me do not get what they deserve"

inddep2: "Others are to blame for the problems people like myself face"

inddep3: "The system works against people like me"

inddep4: "The world is out to get me"

inddep5: "I rarely get what I deserve in life"

System legitimacy and illegitimacy

illeg1: "We live in a sham democracy where a small group secretly controls the country"

illeg2: "Elected governments even in the UK are string puppets that are controlled by hidden forces in the background"

illeg3: "Results in UK elections have been rigged"

illeg4: "The system is rigged to benefit a select few"

illeg5: "Democratic elections in our country are manipulated by powerful elites"

leg1: "The government uses its power legitimately"

leg2: "The government works towards the welfare of people"

leg3: "The government can be trusted to do what is right"

leg4: "In general, the UK political system operates as it should"

leg5: "Most policies serve the greater good"

leg6: "The UK is the best country in the world to live in"

leg7: "The people running the government are corrupt"

distrustpol1: "It does not matter who wins the election in our country because nobody cares about interests of ordinary people anyway"

distrustpol2: "Politicians don't care about the problems of the average person"

distrustpol3: "Politicians more often fight for their own interests than the interests of the whole society"

distrustpol4: “A decent person has no chance to succeed in politics”

sj1: “Society is set up so that people usually get what they deserve”

sj2: “Everyone has a fair shot at wealth and happiness”

sj3: “In general, I find society to be fair”

attcheck2: “Please respond "Strongly disagree" in this row to show that you are paying attention”

—————— Conspiracist ideation, subjectivist relativism, Manicheanism, dichotomous epistemology, distrust in experts, utopianism, actively open-minded thinking ——————

con1: “Most people do not see how much our lives are determined by plots hatched in secret”

con2: “Politicians and other leaders are nothing but the string puppets of powers operating in the background”

con3: “There are certain political circles with secret agendas that are very influential”

con4: “Secret organizations can manipulate people psychologically so that they do not notice how their life is being controlled by others”

subj1: “Truths are simply personal beliefs that indicate how a person sees the world”

subj2: “Truth is a subjective feeling; if it feels correct or obvious to a person then it is true”

subj3: “The truth does not exist – there are only opinions of individual people”

subj4: “What is true depends on what an individual thinks is true”

subj5: “Truth is nothing more than a person’s subjective experience of the world”

mani1: “Life is a battle between good and evil”

mani2: “The conflict between the good and the evil is present everywhere”

mani3: “The forces of good and evil are constantly competing over our thoughts and emotions”

mani4: “The future of humanity depends on the good defeating the evil”

dichotomous1: “An expert who doesn’t come up with a definite answer probably doesn’t know much”

dichotomous2: “If you can’t provide a clear and definite answer to a question, you don’t know what you are talking about”

dichotomous3: “Most problems have only one right answer”

dichotomous4: “There is usually just one single correct interpretation”

distrustexp1: “You can usually rely on the knowledge of those who are recognized as experts” *

distrustexp2: “Those who are called experts are generally overrated”

distrustexp3: “Persons who are considered experts are often frauds”

distrustexp4: “The judgment of the “experts” is often wrong”

utop1: “There is a single, best way to organize a society”

utop2: “With the right politics, an essentially flawless society could be constructed”

utop3: “There is an ideology or worldview that has the solutions to all social problems”

utop4: “There is an ultimate political ideology that would lead to the best society possible”

utop5: “With the right vision, a near perfect society could be constructed”

aot1: “People should always take into consideration evidence that goes against their opinions”

aot2: “It is important to persevere in your opinions even when evidence is brought to bear against them” *

aot3: “Certain opinions are just too important to abandon no matter how good a case can be made against them” *

aot4: “Opinions should always be revised in response to new information or evidence”

aot5: “Loyalty to one’s ideals and principles is more important than “open-mindedness”” *

——— Need for chaos, status-driven risk-taking, collective narcissism, grandiosity, intellectual overconfidence ———

chaos1: “I get a kick when natural disasters strike in foreign countries”

chaos2: “I fantasize about a natural disaster wiping out most of humanity such that a small group of people can start all over”

chaos3: “I think society should be burned to the ground”

chaos4: “When I think about our political and social institutions, I cannot help thinking ‘just let them all burn’”

chaos5: “We cannot fix the problems in our social institutions, we need to tear them down and start over”

chaos6: “I need chaos around me—it is too boring if nothing is going on”

chaos7: “Sometimes I just feel like destroying beautiful things”

status1: “I would rather live as an average person in a safe place than live as a rich and powerful person in a dangerous place” *

status2: “I would enjoy being a famous and powerful person, even if it meant a high risk of assassination

status3: “If I could become rich and famous by winning a major competition, I would put my life on the line to win it”

collnar1: “My group deserves special treatment”

collnar2: “I will never be satisfied until my group gets the recognition it deserves”

collnar3: “It really makes me angry when others criticize my group”

collnar4: “If my group had a major say in the world, the world would be a much better place”

collnar5: “Not many people seem to fully understand the importance of my group”

grand1: “To be honest, I’m just more important than other people”

grand2: “I have outstanding qualities that few others possess”

grand3: “I’m better than almost everyone else”

grand4: “I’ve achieved far more than almost anyone I know”

grand5: “I deserve special treatment”

grand6: “I often have to deal with people who are less important than me”

overcon1: “My ideas are usually better than other people’s ideas”

overcon2: “For the most part, others have more to learn from me than I have to learn from them”

overcon3: “When I am really confident in a belief, there is very little chance that belief is wrong”

overcon4: “I’d rather rely on my own knowledge about most topics than turn to others for expertise”

overcon5: “Listening to perspectives of others seldom changes my mind on important opinions”

attcheck3: “Please respond "Slight agree" in this row to show that you are paying attention”

——— Prejudice against low-status, high-status, least-liked party, and most-liked party groups, and perceptions of antidemocratic attitudes among least-liked and most-liked parties ———

lowstatus1: “Evil”

lowstatus2: “Immoral”

lowstatus3: “Stupid”

lowstatus4: “Savage”

lowstatus5: “Primitive”

lowstatus6: “Barbaric”

highstatus1: “Evil”

highstatus2: “Immoral”

highstatus3: “Stupid”

highstatus4: “Savage”

highstatus5: “Primitive”

highstatus6: “Barbaric”

leastliked1: “Evil”

leastliked2: “Immoral”

leastliked3: “Stupid”

leastliked4: “Savage”

leastliked5: “Primitive”

leastliked6: “Barbaric”

leastlikedperception1: “Political violence can be constructive when it serves the right cause”

leastlikedperception2: “Political messages from some groups should be blocked or censored from the public”

leastlikedperception3: “It is more important that the laws protect our country’s interests than that all individuals have the same rights one hundred percent”

leastlikedperception4: “Whether we live in a democracy or under a dictatorship makes no difference”

leastlikedperception5: “Having a strong leader who is not elected is sometimes better than democracy”

mostliked1: “Evil”

mostliked2: “Immoral”

mostliked3: “Stupid”

mostliked4: “Savage”

mostliked5: “Primitive”

mostliked6: “Barbaric”

mostlikedperception1: “Political violence can be constructive when it serves the right cause”

mostlikedperception2: “Political messages from some groups should be blocked or censored from the public”

mostlikedperception3: “It is more important that the laws protect our country’s interests than that all individuals have the same rights one hundred percent”

mostlikedperception4: “Whether we live in a democracy or under a dictatorship makes no difference”

mostlikedperception5: “Having a strong leader who is not elected is sometimes better than democracy”

Demographic variables

gender: 1 = “Man”, 2 = “Woman”, 3 = “Other”

genderdichotomous: 1 = “Man”, 2 = “Woman”

age: Age in years

education: 1 = “Compulsory school not completed”, 2 = “Compulsory school completed”, 3 = “Upper secondary school completed”, 4 = “University of college studies started”, 5 = “University or colleges studies completed”, 6 = “PhD completed”

leftright: 1 = “Very far to the left”, 2 = “Far to the left”, 3 = “To the left”, 4 = “Slightly to the left”, 5 = “In the middle”, 6 = “Slightly to the right”, 7 = “To the right”, 8 = “Far to the right”, 9 = “Very far to the right”

libcon: 1 = “Extremely liberal”, 2 = “Very liberal”, 3 = “Liberal”, 4 = “Slightly liberal”, 5 = “In the middle”, 6 = “Slightly conservative”, 7 = “Conservative”, 8 = “Very conservative”, 9 = “Extremely conservative”

mostliked: 1 = “Conservative party”, 2 = “Green party”, 3 = “Labour party”, 4 = “Liberal democrats”, 5 = “Scottish National party”, 6 = “UK Independence party”, 7 = “Plaid Cymru”, 8 = “Other”, 9 = “No opinion”

leastliked: 1 = “Conservative party”, 2 = “Green party”, 3 = “Labour party”, 4 = “Liberal democrats”, 5 = “Scottish National party”, 6 = “UK Independence party”, 7 = “Plaid Cymru”, 8 = “Other”, 9 = “No opinion”

religion: 1 = “Christian”, 2 = “Jewish”, 3 = “Muslim”, 4 = “Hindu”, 5 = “Buddhist”, 6 = “Sikh”, 7 = “Atheist”, 8 = “Agnostic”, 9 = “Other”

polactive: “On a scale from 0% to 100%, to what extent do you see yourself as a: - politically active person?”

religiosity: “On a scale from 0% to 100%, to what extent do you see yourself as a: - a religious person?”

Sum scores

AntidemoVdem: The total Vdem score

Elections: Support for restricting democratic elections

Discrimination: Support for discrimination

Censorship: Support for political censorship

Violence: Support for political violence

AntidemoSubscales: Total score for all items included in the four subscales

AntidemoTotal: Total score based on items included in all antidemocratic attitude scales

SDO: Social dominance orientation total

SDOdominance: Group-based dominance

SDOequality: Group-based egalitarianism (reversed)

Authoritarianism: Authoritarianism total of submission and aggression

AuthSubmission: Authoritarian submission

AuthAggression: Authoritarian aggression

GroupAuthoritarianism: Group-based authoritarianism

Breakdown: Perceived breakdown of the social fabric

Cynicism: Cynicism about human nature

DangerousWorld: Belief that the world is dangerous

Apocalypticism: Apocalyptic beliefs

Meritocracy: Belief that society is meritocratic

GroupDeprivation: Group-based deprivation

IndDeprivation: Individual deprivation

SystemIllegitimacy: Perceived illegitimacy of the system

SystemLegitimacy: Perceived legitimacy of the system

SystemJustification: General system justification

DistrustPoliticians: Distrust in politicians

Conspiracism: General conspiracist mindset

Subjectivism: Subjectivist relativism

DichotomousKnowledge: Dichotomous view of knowledge

DistrustExperts: Distrust in experts

Manicheanism: Manichean beliefs

Utopianism: Utopian beliefs

AOT: Actively open-minded thinking

NeedChaos: Need for chaos

StatusRisktaking: Status-driven risk-taking

CollectiveNarcissism: Collective narcissism

Grandiosity: Grandiosity

Overconfidence: Intellectual overconfidence

PrejudiceLowStatus: Prejudice against low-status groups

PrejudiceHighStatus: Prejudice against high-status groups

PrejudiceLeastLiked: Prejudice against people who vote for the least-liked party

PrejudiceMostLiked: Prejudice against people who vote for the least-liked party

PerceptionLeastLiked: Perception of how people voting for the least-liked party would respond

PerceptionMostLiked Perception of how people voting for the most-liked party would respond

ActualScores: Actual scores on the same items

LeastLikedFP: Difference between PerceptionLeastLiked and ActualScores for individuals who chose the participant's least-liked party as their most-liked party

MostLikedFP: Difference between PerceptionMostLiked and ActualScores for individuals who chose the participant's most-liked party as their most-liked party
