# Supplementary material for: The Dark Side of Ideology: Ideological Worldviews and Antidemocratic Attitudes
Source: Ann N Y Acad Sci. 2025 Sep 27;1553(1):391–406. doi: 10.1111/nyas.70062 (PMC12645262; doi:10.1111/nyas.70062)
Supplement: Supplementary file 4 — Supporting information [file NYAS-1553-391-s001.pdf]

Supporting information about the measures

This document contains supplemental information about the measures along with results of confirmatory factor analyses. For a complete list of items included in each scale, see "Codebook.pdf". The measures are organized by sections in the order in which they were included in the survey.

Confirmatory factor analyses (CFA) were run in Jamovi (v. 2.6) with Full Information Maximum Likelihood estimation and fit was evaluated in terms of the Comparative Fit Index (CFI), the Root Mean Square Error of Approximation (RMSEA) with 90% confidence intervals, and the Standardized Root Mean Squared Residual (SRMR). Common ideals are CFI > .95, RMSEA < .06, and SRMR < .08 (Hu & Bentler, 1999). We report sources of model misfit identified through modification indices. According to Kenny (2015), CFA compared to standardized fit criteria will be misleading and should not be reported when RMSEA of the independence model is lower than .158 (because it is mathematically impossible for CFA to satisfy the criteria in these cases).

Contents

- Section 1 (antidemocratic attitudes) 2
- Section 2 (authoritarianism and social dominance orientation) 6
- Section 3 (breakdown, deprivation, cynicism, pessimism, apocalypticism) 9
- Section 4 (perceived system legitimacy/illegitimacy and distrust in politicians) 12
- Section 5 (black-and-white thinking and post-truth attitude) 18
- Section 6 (need for chaos, status-driven dominance, and perceived superiority) 21
- Section 7 (prejudice and false polarization) 24
- Section 8 (demographics) 25
- References 26

Section 1 (antidemocratic attitudes)

Summary

This section included the complete 17-item Claassen et al. (2024) measure of support for liberal democracy. Following Claassen et al. (2024), we calculated a general score based on all items, but we dropped two items ("Unelected authority" and "Judicial review"; items 9 and 12 in the codebook) of eight items that were keyed in the pro-democratic direction because they failed to load on the general factor the scale.

This section also included four scales based on Nilsson's (2024) four-dimensional taxonomy. This scale was originally developed from a larger item pool used in an initial pilot study with data from Swedish adults (N = 147) and then further refined based on data from a representative sample of Swedish adults (N = 2529) with eight items per dimension included.

For this study, we chose the 4-5 best performing items for each dimension. Confirmatory factor analysis testing the four-factor produced good model fit: $\chi^2(146) = 586$ ($p < .001$), CFI = .914, SRMR = .055, RMSEA = .060[.055, .065] ($\lambda \geq .48$). The remaining misfit was due to unmodeled covariation between residuals of some of the censorship items. The correlations between factors ranged from .20 (censorship and violence) to .68 (democratic elections and discrimination). The sum score across all four scales correlated very strongly (r = .75) with the Claassen et al. (2024) V-dem measure of non-democratic values. We therefore computed an aggregated antidemocratic index using items from both scales although three additional items that failed to load on the general factor (the "Civil society" item and two items about censorship; items 5, 27, and 30 in the codebook) had to be removed.

CFA-Output

Factor Loadings

				95% Confidence Interval				
**Factor**	**Indicator**	**Estimate**	**SE**	**Lower**	**Upper**	**Z**	**p**	**Stand. Estimate**
**Elections**	antidemo18	1.305	0.0559	1.195	1.414	23.4	<.001	0.749
	antidemo19	1.137	0.0541	1.030	1.243	21.0	<.001	0.694
	antidemo20	0.902	0.0502	0.804	1.001	18.0	<.001	0.613
	antidemo21	1.154	0.0603	1.036	1.272	19.1	<.001	0.646

Factor Loadings

Factor	Indicator	Estimate	SE	95% Confidence Interval Lower	Upper	Z	p	Stand. Estimate
	antidemo22	1.377	0.0594	1.261	1.493	23.2	<.001	0.747
**Discrimination**	antidemo23	0.893	0.0648	0.766	1.020	13.8	<.001	0.527
	antidemo24	0.824	0.0667	0.693	0.955	12.3	<.001	0.483
	antidemo25	0.877	0.0653	0.749	1.005	13.4	<.001	0.516
	antidemo26	1.079	0.0611	0.959	1.199	17.6	<.001	0.658
**Censorship**	antidemo27	1.163	0.0649	1.035	1.290	17.9	<.001	0.671
	antidemo28	0.898	0.0612	0.778	1.018	14.7	<.001	0.552
	antidemo29	0.883	0.0555	0.774	0.992	15.9	<.001	0.602
	antidemo30	1.101	0.0645	0.975	1.227	17.1	<.001	0.622
	antidemo31	1.057	0.0670	0.926	1.189	15.8	<.001	0.587
**Violence**	antidemo32	1.328	0.0484	1.233	1.423	27.5	<.001	0.826
	antidemo33	1.185	0.0564	1.074	1.295	21.0	<.001	0.681
	antidemo34	0.874	0.0448	0.786	0.962	19.5	<.001	0.646
	antidemo35	1.141	0.0497	1.043	1.238	23.0	<.001	0.730
	antidemo36	1.393	0.0526	1.290	1.496	26.5	<.001	0.805

Factor Covariances

				95% Confidence Interval				
		Estimate	SE	Lower	Upper	Z	p	Stand. Estimate
**Elections**	Elections	1.000[a]						
	Discrimination	0.675	0.0339	0.608	0.742	19.89	<.001	0.675
	Censorship	0.434	0.0406	0.355	0.514	10.70	<.001	0.434
	Violence	0.376	0.0365	0.305	0.448	10.31	<.001	0.376
**Discrimination**	Discrimination	1.000[a]						
	Censorship	0.618	0.0403	0.539	0.697	15.33	<.001	0.618
	Violence	0.332	0.0434	0.247	0.417	7.65	<.001	0.332
**Censorship**	Censorship	1.000[a]						
	Violence	0.204	0.0421	0.121	0.286	4.84	<.001	0.204
**Violence**	Violence	1.000[a]						

[a] fixed parameter

Test for Exact Fit

χ^2	df	p
586	146	<.001

Fit Measures

				RMSEA 90% CI		
CFI	TLI	SRMR	RMSEA	Lower	Upper	AIC
0.913	0.898	0.0561	0.0605	0.0554	0.0657	55763

Section 2 (authoritarianism and social dominance orientation)

Summary

This section included an SDO short-scale (Ho et al., 2015), which covers preference for group-based dominance and hierarchy (half items reversed). It also included short measures of authoritarian submission and aggression (one reversed item in each scale). These were based on a scale developed by Dunwoody and Funke (2016) to reduce extraneous ideological and religious confounds in the measurement of authoritarianism, but we removed a few items due to redundancy and overlap with other scales (e.g., some of the submission items overlap with trust in experts, which we predicted would *reduce* antidemocratic attitudes) and we rephrased one item to make it more clearly authoritarian ("People should never question [original item: 'be critical of'] statements made by individuals in positions of authority"). This section also included three items from a group authoritarianism scale developed by Stellmacher and Petzel (2005), representing the submission, aggression, and conventionalism components respectively.

A CFA-model with SDO, submission, aggression, and group-based authoritarianism as separate factors showed reasonable fit, $\chi^2(146) = 899$ ($p < .001$), CFI = .854, SRMR = .075, RMSEA = .079[.074, .084] ($\lambda \geq .56$, .33, .22, and .47 respectively). The misfit was both due to unmodeled covariation between residuals of some items within scales (particularly submission items) and due to overlap between authoritarianism and SDO.

CFA-Output

Factor Loadings

				**95% Confidence Interval**				
**Factor**	**Indicator**	**Estimate**	**SE**	**Lower**	**Upper**	**Z**	**p**	**Stand. Estimate**
**SDO**	sdo1	1.196	0.0561	1.086	1.306	21.32	<.001	0.692
	sdo2	1.272	0.0559	1.162	1.381	22.75	<.001	0.724
	sdo3	-0.750	0.0452	-0.839	-0.662	-16.59	<.001	-0.563
	sdo4	-0.901	0.0416	-0.983	-0.820	-21.68	<.001	-0.699
	sdo5	1.102	0.0551	0.994	1.210	20.01	<.001	0.655
	sdo6	1.211	0.0529	1.107	1.315	22.90	<.001	0.725

Factor Loadings

Factor	Indicator	Estimate	SE	95% Confidence Interval Lower	Upper	Z	p	Stand. Estimate
	sdo7	-1.012	0.0439	-1.098	-0.926	-23.03	<.001	-0.733
	sdo8	-0.803	0.0411	-0.884	-0.723	-19.53	<.001	-0.647
**Sub-mission**	submission1	1.124	0.0493	1.027	1.221	22.79	<.001	0.781
	submission2	1.069	0.0486	0.973	1.164	21.99	<.001	0.754
	submission3	-0.358	0.0428	-0.442	-0.275	-8.38	<.001	-0.330
	submission4	0.613	0.0419	0.531	0.695	14.64	<.001	0.550
**Aggr-ession**	aggression1	1.132	0.0624	1.009	1.254	18.13	<.001	0.673
	aggression2	1.246	0.0649	1.119	1.373	19.20	<.001	0.717
	aggression3	-0.333	0.0614	-0.453	-0.213	-5.42	<.001	-0.218
	aggression4	1.066	0.0697	0.930	1.203	15.30	<.001	0.585
**Group author-itarianism**	groupauth1	1.140	0.0602	1.022	1.258	18.94	<.001	0.683
	groupauth2	0.752	0.0619	0.630	0.873	12.14	<.001	0.472
	groupauth3	1.267	0.0578	1.154	1.380	21.92	<.001	0.794

Test for Exact Fit

χ^2	df	p
899	146	<.001

Fit Measures

				RMSEA 90% CI	
**CFI**	**TLI**	**SRMR**	**RMSEA**	**Lower**	**Upper**
0.854	0.829	0.0751	0.0793	0.0744	0.0843

Section 3 (breakdown of social fabric, deprivation, cynicism, pessimism, apocalypticism)

Summary

This section contained the breakdown of social fabric dimension of anomie (Teymoori et al., 2016), but two items were excluded due to overlap with cynicism. It contained a group-based relative deprivation scale with items from Obaidi et al. (2019) that we rephrased so that they would be applicable to people in general rather than just Muslims (e.g., “I think that people in my group [Muslims] are disadvantaged because society [the West] oppresses them”). It also contained three items drawn from Armaly and Enders (2024, e.g., “The world is out to get me”) and two from Berntzen et al. (2024, e.g., “Others are to blame for the problems people like myself face”) to measure an individual sense of deprivation. Finally, this section included short scales measuring cynicism, dangerous world belief (two reversed items), apocalypticism, and belief that society is meritocratic from a recent large-scale effort to integrate measures of people’s most fundamental beliefs about the world (Nilsson & Bäckström, 2024).

A model with all scales represented by separate factors showed good fit, $\chi^2(356) = 1170$ ($p <$ .001), CFI = .926, SRMR = .053, RMSEA = .053[.050, .056] ($\lambda \geq .47$). The misfit was mainly due to unmodeled covariation between dangerous world items and other scales.

CFA-Output

Factor Loadings

				95% Confidence Interval				
**Factor**	**Indicator**	**Estimate**	**SE**	**Lower**	**Upper**	**Z**	**p**	**Stand. Estimate**
**Breakdown social fabric**	breakdown1	0.871	0.0536	0.766	0.976	16.3	<.001	0.574
	breakdown2	0.868	0.0537	0.762	0.973	16.2	<.001	0.568
	breakdown3	0.860	0.0572	0.748	0.972	15.0	<.001	0.533
	breakdown4	1.023	0.0472	0.931	1.115	21.7	<.001	0.720
**Cynicism**	cynicism1	0.879	0.0435	0.794	0.964	20.2	<.001	0.668
	cynicism2	1.071	0.0487	0.976	1.167	22.0	<.001	0.712
	cynicism3	1.005	0.0433	0.920	1.090	23.2	<.001	0.743

Factor Loadings

				95% Confidence Interval				
**Factor**	**Indicator**	**Estimate**	**SE**	**Lower**	**Upper**	**Z**	**p**	**Stand. Estimate**
	cynicism4	0.976	0.0440	0.890	1.063	22.2	<.001	0.718
**Dangerous world**	dang1	1.315	0.0476	1.222	1.409	27.6	<.001	0.843
	dang2	-1.064	0.0450	-1.152	-0.976	-23.6	<.001	-0.754
	dang3	0.764	0.0582	0.650	0.878	13.1	<.001	0.468
	dang4	-1.201	0.0472	-1.293	-1.108	-25.4	<.001	-0.796
**Apocalyp-ticism**	apoc1	0.863	0.0445	0.776	0.951	19.4	<.001	0.643
	apoc2	1.280	0.0537	1.175	1.385	23.9	<.001	0.750
	apoc3	1.356	0.0497	1.258	1.453	27.3	<.001	0.828
	apoc4	1.292	0.0474	1.199	1.385	27.2	<.001	0.827
**Meritocracy**	merit1	1.119	0.0450	1.031	1.207	24.9	<.001	0.785
	merit2	1.107	0.0473	1.014	1.200	23.4	<.001	0.749
	merit3	1.043	0.0441	0.957	1.129	23.7	<.001	0.756
	merit4	1.133	0.0466	1.042	1.224	24.3	<.001	0.771
**Group deprivation**	groupdep1	1.503	0.0513	1.402	1.603	29.3	<.001	0.856
	groupdep2	1.324	0.0517	1.223	1.425	25.6	<.001	0.782
	groupdep3	1.481	0.0542	1.375	1.587	27.3	<.001	0.817
	groupdep4	0.980	0.0545	0.874	1.087	18.0	<.001	0.602

Factor Loadings

				95% Confidence Interval				
**Factor**	**Indicator**	**Estimate**	**SE**	**Lower**	**Upper**	**Z**	**p**	**Stand. Estimate**
**Individual deprivation**	inddep1	1.281	0.0470	1.189	1.373	27.2	<.001	0.821
	inddep2	0.856	0.0530	0.752	0.960	16.1	<.001	0.549
	inddep3	1.352	0.0506	1.253	1.451	26.7	<.001	0.810
	inddep4	0.789	0.0471	0.697	0.881	16.8	<.001	0.567
	inddep5	1.109	0.0483	1.015	1.204	23.0	<.001	0.732

Test for Exact Fit

χ^2	df	p
1170	356	<.001

Fit Measures

				RMSEA 90% CI	
**CFI**	**TLI**	**SRMR**	**RMSEA**	**Lower**	**Upper**
0.926	0.916	0.0529	0.0529	0.0495	0.0563

Section 4 (perceived system legitimacy/illegitimacy and distrust in politicians)

Summary

This section was informed by a pilot study ($N = 80$). It contained seven items measuring perceived system legitimacy, including three from the general system justification scale (e.g., "Most policies serve the greater good", Kay & Jost, 2003), two from the breakdown of leadership dimension of anomie (e.g., "The government uses its power legitimately", Teymoori et al., 2016), and two items about trust ("The government can be trusted to do what is right") and corruption ("The people running the government are corrupt") inspired by Armaly and Enders (2024). It also contained five items measuring perceived system illegitimacy, including four from a measure of conspiracy theories about the system (e.g., We live in a sham democracy where a small group secretly controls the country") and one item ("The system is rigged to benefit a select few") from Armaly and Enders (2024), and four items measuring distrust in politicians (three from Šerek & Mužík, 2021 and one from Teymoori et al., 2016).

The item about corruption in the government was subsequently dropped due to cross-loadings on other factors. A three-factor model showed excellent fit, $\chi^2(87) = 338$ ($p < .001$), CFI = .963, SRMR = .041, RMSEA = .060[.053, .066] ($\lambda \geq .48$; see CFA-output with and without the corruption item below). Separate scales were therefore retained for system legitimacy, system illegitimacy, and distrust in politicians. The correlation between the legitimacy and illegitimacy factors was .68. This section also contained three additional items from the system justification scale that are more related to meritocratic world belief (Kay & Jost, 2003, e.g., "Everyone has a fair shot at wealth and happiness"; which allowed us to use system justification (six items) as an alternative mediator (i.e., tree items included in the system legitimacy and system justification scales were the same). The two remaining items in the system justification were excluded due to overlap with other scales. Following the pre-registration, we also calculated an aggregated system legitimacy score with the illegitimacy items as reversed items ($\alpha = .90$, $\omega_h = .69$, $\omega_t = .93$), but we present results based on legitimacy and illegitimacy as separate scales as these analyses presented a clearer and more nuanced picture.

CFA-Output

Factor Loadings

				95% Confidence Interval				
**Factor**	**Indicator**	**Estimate**	**SE**	**Lower**	**Upper**	**Z**	**p**	**Stand. Estimate**
**System illegitimacy**	illeg1	1.591	0.0543	1.484	1.697	29.3	<.001	0.852
	illeg2	1.502	0.0527	1.398	1.605	28.5	<.001	0.837

Factor Loadings

				95% Confidence Interval				
**Factor**	**Indicator**	**Estimate**	**SE**	**Lower**	**Upper**	**Z**	**p**	**Stand. Estimate**
	illeg3	1.132	0.0507	1.032	1.231	22.3	<.001	0.705
	illeg4	1.123	0.0520	1.021	1.225	21.6	<.001	0.691
	illeg5	1.341	0.0520	1.239	1.443	25.8	<.001	0.783
**System legitimacy**	leg1	1.225	0.0457	1.136	1.315	26.8	<.001	0.800
	leg2	1.204	0.0434	1.119	1.289	27.8	<.001	0.819
	leg3	1.177	0.0448	1.089	1.265	26.3	<.001	0.788
	leg4	1.194	0.0490	1.098	1.290	24.4	<.001	0.749
	leg5	0.989	0.0433	0.904	1.074	22.8	<.001	0.715
	leg6	0.773	0.0580	0.659	0.886	13.3	<.001	0.460
	leg7	-1.300	0.0499	-1.398	-1.202	-26.1	<.001	-0.787
**Distrust politicians**	distrustpol1	1.303	0.0574	1.190	1.415	22.7	<.001	0.721
	distrustpol2	1.334	0.0468	1.243	1.426	28.5	<.001	0.847
	distrustpol3	0.974	0.0431	0.889	1.058	22.6	<.001	0.720
	distrustpol4	1.026	0.0555	0.917	1.135	18.5	<.001	0.618

Factor Covariances

		Estimate	SE	95% Confidence Interval		Z	p	Stand. Estimate
				Lower	Upper			
**System illegitimacy**	System illegitimacy	1.000[a]						
	System legitimacy	-0.733	0.0210	-0.774	-0.692	-35.0	<.001	-0.733
	Distrust politicians	0.778	0.0203	0.738	0.818	38.3	<.001	0.778
**System legitimacy**	System legitimacy	1.000[a]						
	Distrust politicians	-0.836	0.0168	-0.869	-0.803	-49.9	<.001	-0.836
**Distrust politicians**	Distrust politicians	1.000[a]						

[a] fixed parameter

Test for Exact Fit

χ^2	df	p
584	101	<.001

Fit Measures

				RMSEA 90% CI	
**CFI**	**TLI**	**SRMR**	**RMSEA**	**Lower**	**Upper**
0.937	0.925	0.0514	0.0767	0.0708	0.0828

Factor Loadings

				95% Confidence Interval				
**Factor**	**Indicator**	**Estimate**	**SE**	**Lower**	**Upper**	**Z**	**p**	**Stand. Estimate**
**System illegitimacy**	illeg1	1.594	0.0542	1.487	1.700	29.4	<.001	0.854
	illeg2	1.502	0.0527	1.399	1.606	28.5	<.001	0.837
	illeg3	1.129	0.0508	1.029	1.228	22.2	<.001	0.704
	illeg4	1.121	0.0521	1.019	1.223	21.5	<.001	0.689
	illeg5	1.341	0.0521	1.239	1.443	25.8	<.001	0.783
**System legitimacy**	leg1	1.231	0.0458	1.141	1.321	26.9	<.001	0.804
	leg2	1.228	0.0432	1.143	1.312	28.5	<.001	0.835
	leg3	1.186	0.0449	1.098	1.275	26.4	<.001	0.795
	leg4	1.198	0.0493	1.101	1.294	24.3	<.001	0.751
	leg5	1.017	0.0431	0.932	1.101	23.6	<.001	0.735
	leg6	0.798	0.0580	0.684	0.912	13.7	<.001	0.475
**Distrust politicians**	distrustpol1	1.307	0.0575	1.195	1.420	22.8	<.001	0.724
	distrustpol2	1.332	0.0470	1.240	1.424	28.3	<.001	0.846

Factor Loadings

				95% Confidence Interval				
**Factor**	**Indicator**	**Estimate**	**SE**	**Lower**	**Upper**	**Z**	**p**	**Stand. Estimate**
	distrustpol3	0.973	0.0432	0.888	1.057	22.5	<.001	0.719
	distrustpol4	1.027	0.0556	0.918	1.136	18.5	<.001	0.618

Factor Covariances

				95% Confidence Interval				
		**Estimate**	**SE**	**Lower**	**Upper**	**Z**	**p**	**Stand. Estimate**
**System illegitimacy**	System illegitimacy	1.000[a]						
	System legitimacy	-0.675	0.0237	-0.722	-0.629	-28.5	<.001	-0.675
	Distrust politicians	0.778	0.0203	0.738	0.818	38.3	<.001	0.778
**System legitimacy**	System legitimacy	1.000[a]						
	Distrust politicians	-0.795	0.0190	-0.832	-0.758	-41.9	<.001	-0.795
**Distrust politicians**	Distrust politicians	1.000[a]						

[a] fixed parameter

Factor Covariances

	Estimate	SE	95% Confidence Interval		Z	p	Stand. Estimate
			Lower	Upper			

Test for Exact Fit

χ^2	df	p
338	87	<.001

Fit Measures

CFI	TLI	SRMR	RMSEA	RMSEA 90% CI	
				Lower	Upper
0.963	0.955	0.0410	0.0596	0.0530	0.0664

Section 5 (black-and-white thinking and post-truth attitude)

Summary

This section covered epistemological beliefs mainly based on Nilsson and Bäckström's (2024) large-scale integration of belief scales. Scales relating to black-and-white thinking included a dichotomous view of knowledge, Manicheanism, and utopianism. Scales relating to a post-truth attitude were subjectivist relativism (see also Aspernäs et al., 2023), conspiracist mindset, and distrust in experts (one reversed item). The post-truth category also included a short-measure of actively open-minded thinking (Pennycook et al., 2020, who rephrased some items slightly to remove religious connotations).

A model with all epistemology scales as separate factors showed good fit, $\chi^2(413) = 1567$ ($p <$ .001), CFI = .911, SRMR = .056, RMSEA = .059[.056, .062] ($\lambda \geq .38$ for AOT and .55 for all other scales). The remaining misfit was mostly due to unmodeled covariation between residuals of single items within the dichotomous knowledge and utopianism scales.

CFA-Output

Factor Loadings

				95% Confidence Interval				
**Factor**	**Indicator**	**Estimate**	**SE**	**Lower**	**Upper**	**Z**	**p**	**Stand. Estimate**
**Conspiracist ideation**	con1	1.480	0.0529	1.376	1.584	28.0	<.001	0.832
	con2	1.519	0.0524	1.416	1.621	29.0	<.001	0.850
	con3	1.151	0.0498	1.053	1.249	23.1	<.001	0.728
	con4	1.486	0.0551	1.378	1.594	27.0	<.001	0.810
**Subjectivism**	subj1	1.365	0.0495	1.268	1.462	27.6	<.001	0.821
	subj2	1.313	0.0497	1.215	1.410	26.4	<.001	0.798
	subj3	1.136	0.0510	1.035	1.236	22.2	<.001	0.707
	subj4	1.371	0.0532	1.267	1.476	25.8	<.001	0.786
	subj5	1.367	0.0488	1.272	1.463	28.0	<.001	0.829

Factor Loadings

				95% Confidence Interval				
**Factor**	**Indicator**	**Estimate**	**SE**	**Lower**	**Upper**	**Z**	**p**	**Stand. Estimate**
**Manichean-ism**	mani1	1.471	0.0494	1.374	1.568	29.8	<.001	0.866
	mani2	1.302	0.0474	1.209	1.395	27.5	<.001	0.821
	mani3	1.390	0.0491	1.294	1.487	28.3	<.001	0.839
	mani4	1.120	0.0504	1.021	1.219	22.2	<.001	0.706
**Dichotomous knowledge**	dichotomous1	1.167	0.0530	1.063	1.271	22.0	<.001	0.758
	dichotomous2	1.082	0.0577	0.969	1.195	18.7	<.001	0.656
	dichotomous3	0.857	0.0516	0.756	0.958	16.6	<.001	0.606
	dichotomous4	0.838	0.0517	0.736	0.939	16.2	<.001	0.599
**Distrust experts**	distrustexp1	0.759	0.0470	0.667	0.852	16.2	<.001	0.554
	distrustexp2	-1.326	0.0462	-1.417	-1.235	-28.7	<.001	-0.855
	distrustexp3	-1.241	0.0460	-1.331	-1.151	-27.0	<.001	-0.822
	distrustexp4	-1.095	0.0455	-1.184	-1.006	-24.1	<.001	-0.756
**Utopianism**	utop1	0.966	0.0489	0.870	1.061	19.7	<.001	0.659
	utop2	1.285	0.0515	1.184	1.386	24.9	<.001	0.788
	utop3	1.092	0.0523	0.989	1.194	20.9	<.001	0.691
	utop4	1.179	0.0484	1.084	1.274	24.4	<.001	0.767

Factor Loadings

				95% Confidence Interval				
**Factor**	**Indicator**	**Estimate**	**SE**	**Lower**	**Upper**	**Z**	**p**	**Stand. Estimate**
**AOT**	utop5	1.267	0.0517	1.166	1.368	24.5	<.001	0.778
	aot1	0.429	0.0427	0.345	0.512	10.0	<.001	0.382
	aot2	-1.170	0.0548	-1.278	-1.063	-21.4	<.001	-0.721
	aot3	-1.041	0.0558	-1.150	-0.932	-18.7	<.001	-0.648
	aot4	0.462	0.0434	0.377	0.547	10.6	<.001	0.403
	aot5	-1.060	0.0515	-1.161	-0.959	-20.6	<.001	-0.699

Test for Exact Fit

χ^2	df	p
1567	413	<.001

Fit Measures

				RMSEA 90% CI	
**CFI**	**TLI**	**SRMR**	**RMSEA**	**Lower**	**Upper**
0.911	0.900	0.0563	0.0587	0.0557	0.0618

Section 6 (need for chaos, status-driven dominance, and perceived superiority)

Summary

This section measured need for chaos (Arceneaux et al., 2021, status-driven risk-taking (Ashton et al., 2010), collective narcissism (Golec de Zavala et al., 2009), grandiosity (the PID-5; Krueger et al., 2012), and intellectual overconfidence (Krumrei-Mancuso et al., 2016).

A model with all scales represented by separate factors had acceptable fit, $\chi^2(289) = 1564$, < .001, CFI = .864, SRMR = .064, RMSEA = .074[.070, .078] ($\lambda \geq .48$). The correlations between the factors were strong (e.g., need for chaos correlated ≥ .61 with all other factors and the superiority factors correlated between .71 and .88. The misfit was caused primarily by unmodeled cross-loadings between collective narcissism and the other superiority scales and between need for chaos and perceived superiority.

CFA-Output

Factor Loadings

				95% Confidence Interval				
**Factor**	**Indicator**	**Estimate**	**SE**	**Lower**	**Upper**	**Z**	**p**	**Stand. Estimate**
**Need for chaos**	chaos1	0.688	0.0457	0.598	0.778	15.0	<.001	0.540
	chaos2	0.983	0.0493	0.886	1.079	19.9	<.001	0.669
	chaos3	1.013	0.0463	0.922	1.104	21.9	<.001	0.724
	chaos4	1.112	0.0576	0.999	1.225	19.3	<.001	0.659
	chaos5	0.919	0.0602	0.801	1.037	15.3	<.001	0.544
	chaos6	0.719	0.0464	0.629	0.810	15.5	<.001	0.547
	chaos7	0.719	0.0387	0.643	0.795	18.6	<.001	0.635
**Status risk-taking**	status1	0.694	0.0550	0.586	0.802	12.6	<.001	0.476
	status2	-1.138	0.0522	-1.241	-1.036	-21.8	<.001	-0.774
	status3	-1.275	0.0593	-1.391	-1.159	-21.5	<.001	-0.763
**Collective narcissism**	collnar1	1.079	0.0459	0.989	1.169	23.5	<.001	0.770
	collnar2	1.172	0.0544	1.065	1.278	21.6	<.001	0.713
	collnar3	1.000	0.0614	0.880	1.120	16.3	<.001	0.576
	collnar4	1.040	0.0583	0.925	1.154	17.8	<.001	0.612
	collnar5	1.217	0.0535	1.112	1.321	22.8	<.001	0.740
**Grandiosity**	grand1	1.019	0.0394	0.942	1.096	25.9	<.001	0.786
	grand2	1.187	0.0523	1.085	1.290	22.7	<.001	0.716

Factor Loadings

Factor	Indicator	Estimate	SE	95% Confidence Interval Lower	Upper	Z	p	Stand. Estimate
	grand3	1.034	0.0391	0.957	1.110	26.5	<.001	0.798
	grand4	1.046	0.0477	0.953	1.140	21.9	<.001	0.698
	grand5	1.031	0.0426	0.947	1.114	24.2	<.001	0.751
	grand6	1.045	0.0483	0.950	1.140	21.6	<.001	0.690
**Overconfidence**	overcon1	1.188	0.0490	1.092	1.284	24.2	<.001	0.764
	overcon2	1.083	0.0465	0.992	1.174	23.3	<.001	0.743
	overcon3	1.040	0.0516	0.939	1.141	20.2	<.001	0.669
	overcon4	0.884	0.0531	0.780	0.988	16.7	<.001	0.573
	overcon5	0.816	0.0535	0.711	0.921	15.2	<.001	0.534

Factor Covariances

		Estimate	SE	95% Confidence Interval Lower	Upper	Z	p	Stand. Estimate
**Need for chaos**	Need for chaos	1.000[a]						
	Status risk-taking	-0.610	0.0331	-0.675	-0.545	-18.4	<.001	-0.610
	Collective narcissism	0.607	0.0308	0.547	0.667	19.7	<.001	0.607
	Grandiosity	0.659	0.0272	0.606	0.713	24.2	<.001	0.659
	Overconfidence	0.617	0.0307	0.557	0.677	20.1	<.001	0.617
**Status risk-taking**	Status risk-taking	1.000[a]						
	Collective narcissism	-0.454	0.0388	-0.530	-0.377	-11.7	<.001	-0.454
	Grandiosity	-0.624	0.0302	-0.684	-0.565	-20.7	<.001	-0.624
	Overconfidence	-0.531	0.0359	-0.601	-0.460	-14.8	<.001	-0.531
**Collective narcissism**	Collective narcissism	1.000[a]						
	Grandiosity	0.713	0.0262	0.661	0.764	27.2	<.001	0.713

Factor Covariances

				95% Confidence Interval				
		**Estimate**	**SE**	**Lower**	**Upper**	**Z**	**p**	**Stand. Estimate**
**Grandiosity**	Overconfidence	0.727	0.0261	0.676	0.778	27.9	<.001	0.727
	Grandiosity	1.000[a]						
	Overconfidence	0.881	0.0159	0.849	0.912	55.4	<.001	0.881
**Overconfidence**	Overconfidence	1.000[a]						

[a] fixed parameter

Test for Exact Fit

χ^2	df	p
1564	289	<.001

Fit Measures

				RMSEA 90% CI	
**CFI**	**TLI**	**SRMR**	**RMSEA**	**Lower**	**Upper**
0.864	0.847	0.0640	0.0740	0.0704	0.0776

Section 7 (prejudice and false polarization)

Summary

In Section 7, the participants completed the same six items for low-status and high-status groups ("Please think about poor and powerless people at the bottom of society [rich and powerful people at the top of society]) and for least-liked and most-liked party ("Please think about people who vote for the political party you like the least, that is your political opponents [the political party you like the most, that is your political allies]"), rating how much these groups are "evil", "immoral", "stupid", "savage", "primitive", and "barbaric". The selection of attributes was inspired by several recent papers (Costello et al., 2022; Finkel et al., 2024; Landry et al., 2023). The participants were also asked to indicate how they thought people who vote for the parties they like the least and the most would respond to five items representing general antidemocratic attitudes and the four subdimensions ("Please indicate how much you think that an average person in this group would agree or disagree with the following statements, that is how your political opponents [allies] would respond").

Section 8 (demographics)

Summary

This section included two additional questions in addition to the items we used in the analyses. The first was about ingroup identification: “When you think about "your group", which kind of group do you mainly think of? Check all that apply.” (“A political group”, “A religious group”, “An ethnic group”, “A national group”, “Gender or age group”, “Family or friends”, “Occupational group”, “Other”). The second was about perceived threats: “To what extent do you see the following as major threats currently facing us?” (“Climate catastrophe”, “Democratic collapse”, “Terrorism”, “War”, “Economic collapse”, “Racism”, “Inequality”, “Natural disasters”, “Multiculturalism”, “Criminality”). The participants rated the threats on Likert scales ranging from 1 (“Not at all”) to 7 (“Extremely”). We sought to include a variety of perceived threats plausibly associated with different ideological orientations.

References

Arceneaux, K., Gravelle, T. B., Osmundsen, M., Bang Petersen, M., Reifler, J., & Scotto, T. J. (2021). Some people just want to watch the world burn: The prevalence, psychology and politics of the 'Need for Chaos'. *Philosophical Transactions of the Royal Society B*, *376(*1822): 20200147. https://doi.org/10.1098/rstb.2020.0147

Armaly, M. T. & Enders, A. M. (2024). Who supports political violence? *Perspectives on Politics, 22*(2), 427-444. https://doi.org/10.1017/S1537592722001086

Ashton, M. C., Lee, K., Pozzebon, J. A., Visser, B. A., & Worth, N. C. (2010). Status-driven risk taking and the major dimensions of personality. *Journal of Research in Personality, 44*(6), 734–737. https://doi.org/10.1016/j.jrp.2010.09.003

Berntzen, L. E. N., Kelsall, H., & Harteveld, E. (2024). Consequences of affective polarization: Avoidance, intolerance and support for violence in the United Kingdom and Norway. *European Journal of Political Research, 63*(3), 927–949. https://doi.org/10.1111/1475-6765.12623

Claassen, C., Ackermann, K., Bertsou, E., Borba, L., Carlin, R. E., Cavari, A., Dahlum, S., Gherghina, S., Hawkins, D., Lelkes, Y., Magalhães, P. C., Mattes, R., Meijers, M. J., Neundorf, A., Oross, D., Öztürk, A., Sarsfield, R., Self, D., Stanley, B., … Zechmeister, E. J. (2024). Conceptualizing and measuring support for democracy: A new approach. *Comparative Political*. https://doi.org/10.1177/00104140241259458

Finkel, E., Landry, A., Hoyle, R. H., Druckman, J., & Van Bavel, J. J. (2024, January 25). Partisan antipathy and the erosion of democratic norms. https://doi.org/10.31234/osf.io/ahgy6

Golec de Zavala, A., Cichocka, A., Eidelson, R., & Jayawickreme, N. (2009). Collective narcissism and its social consequences. *Journal of Personality and Social Psychology*, *97*, 1074-1096. https://doi.org/10.1037/a0016904

Ho, A. K., Sidanius, J., Kteily, N., Sheehy-Skeffington, J., Pratto, F., Henkel, K. E., Foels, R., & Stewart, A. L. (2015). The nature of social dominance orientation: Theorizing and measuring preferences for intergroup inequality using the new SDO_7 scale. *Journal of Personality and Social Psychology*, *109*(6):1003-1028. https://doi.org/10.1037/pspi0000033

Kay, A. C., & Jost, J. T. (2003). Complementary justice: Effects of "poor but happy" and "poor but honest" stereotype exemplars on system justification and implicit activation of the justice motive. *Journal of Personality and Social Psychology*, *85*(5), 823-837. https://doi.org/10.1037/0022-3514.85.5.823

Kenny, D. A. (2024, October 6). Measuring model fit. https://davidkenny.net/cm/fit.htm

Krueger, R. F., Derringer, J., Markon, K. E., Watson, D., & Skodol, A. E. (2012). Initial construction of a maladaptive personality trait model and inventory for DSM-5. *Psychological Medicine*, 42(9), 1879-1890. https://doi.org/10.1017/S0033291711002674

Krumrei-Mancuso, E. J. & Rouse, S. V. (2016). The development and validation of the comprehensive intellectual humility scale. *Journal of Personality Assessment*, *98*(2), 209-210. https://doi.org/10.1080/00223891.2015.1068174

Landry, A. P., Schooler, J. W., Willer, R., & Seli, P. (2023). Reducing explicit blatant dehumanization by correcting exaggerated meta-perceptions. *Social Psychological and Personality Science, 14*(4), 407–418. https://doi.org/10.1177/19485506221099146

Nilsson, A. (2024). Antidemocratic tendencies on the left, the right, and beyond: A critical review of the theory and measurement of left-wing authoritarianism. *Political Psychology*. https://onlinelibrary.wiley.com/doi/abs/10.1111/pops.12951

Nilsson, A. & Bäckström, M. (2024, April 3). *The structure of basic beliefs about the world* [Paper presentation]. World conference of personality, Willemstad, Curaçao.

Obaidi, M., Bergh, R., Akrami, N., & Anjum, G. (2019). Group-based relative deprivation explains endorsement of extremism among Western-born Muslims. *Psychological Science*, *30*(4), 596-605. https://doi.org/10.1177/0956797619834879

Pennycook, G., Cheyne, J. A., Koehler, D. J., & Fugelsang, J. A. (2020). On the belief that beliefs should change according to evidence: Implications for conspiratorial, moral, paranormal, political, religious, and science beliefs. *Judgment and Decision Making, 15*(4), 476-498. https://doi.org/10.1017/S1930297500007439

Šerek, J., & Mužík, M. (2021). Who does *not* protect democracy?: Examining the role of right-wing authoritarianism and social dominance orientation. *Personality and Individual Differences, 181:* 111027. https://doi.org/10.1016/j.paid.2021.111027

Stellmacher, J., & Petzel, T. (2005). Authoritarianism as a group phenomenon. *Political Psychology, 26*(2), 245–274. https://doi.org/10.1111/j.1467-9221.2005.00417.x

Teymoori, A., Jetten, J., Bastian, B., Ariyanto, A., Autin, F., Ayub, N., Badea, C., Besta, T., Butera, F., Costa-Lopes, R., Cui, L., Fantini, C., Finchilescu, G., Gaertner, L., Gollwitzer, M., Gómez, Á., González, R., Hong, Y. Y., Jensen, D. H., . . . Wohl, M. (2016). Revisiting the measurement of anomie. *PLoS ONE, 11*(7): e0158370. https://doi.org/10.1371/journal.pone.0158370
